# Artificial Intelligence in Digital Self-Diagnosis Tools: A Narrative Overview of Reviews

**DOI:** 10.1016/j.mcpdig.2025.100242

**Published:** 2025-06-10

**Authors:** Aikaterini Mentzou, Amy Rogers, Edzia Carvalho, Angela Daly, Maeve Malone, Xaroula Kerasidou

**Affiliations:** a Division of Psychology, School of Humanities, Social Sciences and Law, University of Dundee, UK; b Division of Cardiovascular Medicine, School of Medicine, University of Dundee, UK; c Politics and International Relations, School of Humanities, Social Sciences and Law, University of Dundee, UK; d Leverhulme Research Centre for Forensic Science, University of Dundee, UK; e Dundee Law School, School of Humanities, Social Sciences and Law, University of Dundee, UK; f Ethox Centre, Oxford Population Health (Nuffield Department of Population Health), Big Data Institute, Li Ka Shing Centre for Health Information and Discovery Oxford, University of Oxford, UK

## Abstract

Digital self-diagnosis tools, or symptom checkers, many of which incorporate artificial intelligence technology, are intended to provide diagnostic information and triage advice to lay users. This narrative overview of reviews explores the common themes and issues raised by existing evidence synthesis literature on these tools to establish a common ground for interdisciplinary research. We searched 3 bibliographic databases (PubMed, Scopus and Web of Science) and Google Scholar using keyword combinations of *artificial*, *self-diagnosis*, *intelligence*, and *machine learning* for publications from 2019 to 2023. We included systematic reviews, meta-analyses, scoping reviews, narrative syntheses, and opinion pieces that discussed tools where users proactively entered personal health information to acquire a predicted diagnosis of their symptoms or triage advice. This overview reveals significant gaps in understanding the key areas of development, implementation, impact, and oversight of digital self-diagnosis tools. Additionally, the terminology used to describe these tools and their underlying technologies varies widely, encompassing technologies ranging from simple branching logic algorithms to complex deep neural networks. Our interdisciplinary analysis identified gaps and critical areas for future research across all stages of the lifecycles of these tools. The diverse challenges uncovered highlight the necessity for multiagency and multidisciplinary efforts promoting responsible development and implementation.

**Article Highlights**

• This is a narrative review, from an interdisciplinary perspective, of research analyzing the use of digital self-diagnosis tools, including those that use artificial intelligence technology.
• Findings highlight significant gaps in understanding the key areas of development, implementation, impact, and oversight of digital self-diagnosis tools.
• Findings also note the lack of clarity in the understanding and use of the terminology used to describe these tools and their underlying technologies.

In our digital age, technology mediates everyday encounters, including health information-seeking behavior.^1^^,^^2^ Many people use online resources before medical appointments,^1^ with healthcare–related searches accounting for 7% of Google queries—over 1 billion daily.^3^ An US study found that internet use for health information increased from 61.2% in 2008 to 74.4% in 2017.^4^

With global healthcare systems facing staff shortages, increased demand, and long waiting times, applying technology to healthcare is often presented as a necessity.^5^ This imperative is supported by policy and driven by cost reduction and primary care accessibility goals. The UK National Health Service (NHS) 111 platform exemplifies this approach, aiming to reduce frontline healthcare demand and improve triage.^6^^,^^7^ The past 2 decades have seen rapid growth in digital healthcare tools, often powered by artificial intelligence (AI), including self-diagnosis symptom checkers providing users with medical information and triage advice.^8^

We conducted a comprehensive literature search on AI and health using Google Scholar (April to June 2023). Our findings reveal a significant increase in AI and digital healthcare research since 2019, along with diverse terminology for self-diagnosis tools. Given the volume, terminological heterogeneity, and potential disciplinary silos (eg, medicine and computing), we argue for an interdisciplinary interpretation of existing reviews.

This narrative overview of reviews examines key themes in evidence synthesis literature on AI-powered digital self-diagnosis tools using an interdisciplinary approach. Table 1 defines the technological terms used.

**Table 1:** Definitions of Terms Used in This Article

Term	Definition
Algorithm	A set of instructions to execute a task, used in this article to refer exclusively to software algorithms or programs
Artificial intelligence (AI)	A broad term encompassing many computational approaches that aim to produce a computer that can think and learn like a human
Digital healthcare tool	Software designed to accomplish a health-related goal; includes tools designed for lay users and trained healthcare staff
Digital self-diagnosis tool	Software that provides lay users with diagnostic information and advice when they enter personal health information (eg, symptoms, signs)
Machine learning	An AI approach where a computer is taught to learn from experience without being explicitly programmed with knowledge
Machine learning model	A type of AI algorithm that has learned to recognize patterns in data and can make predictions or decisions based on new data inputs
Natural language processing	A type of AI that combines computational linguistics and machine learning to understand and generate human language (eg, chatbots, machine translation, and speech recognition)
Neural networks	A powerful type of machine learning model inspired by the structure of the human brain

### Medical Diagnosis

For much of recorded history, diagnosing human illness has been considered the preserve of traditional healers and medical doctors, who draw on, sometimes arcane, knowledge and professional experience. However, in 1959, Ledley and Lusted^9^ conceptualized the cognitive process of diagnosis as one of probability, based on Bayes' theorem. This mathematical approach opened the theoretical possibility of using computing to accomplish the human activity of diagnosis.

The appearance of computer-based clinical diagnostic tools in the late 20th century coincided with an acceleration in the field of Informatics.^10^ Currently available computerized clinical decision support (CDS) systems, often embedded in healthcare administrative systems, use individual patients’ symptoms, signs, and test results to generate recommendations to support clinicians’ decision making process.^10^ Many CDS systems use knowledge-based branching logic algorithms, meaning they are manually coded using existing medical knowledge, such as published clinical guidelines.^11^ However, CDS systems increasingly use AI approaches such as machine learning to generate recommendations based on large volumes of routinely collected clinical data.^10^ This use of machine learning algorithms has been criticized as reducing professional epistemic authority and changing norms around risk management towars risk-averse decision making.^12^

In contrast to CDS systems, which aim to augment clinical care delivery by supporting a doctor’s decision making skills, a new generation of software tools is aimed directly at patients as consumers. Symptom checker apps and digital triage tools collect data directly from individuals and generate possible diagnoses and advice. Many of these tools are knowledge-based systems, such as the UK NHS 111 symptom checker (https://111.nhs.uk/triage/check-your-symptoms). However, more complex AI-based self-diagnosis tools that claim to use deep machine learning techniques, such as neural networks, are also proliferating.^8^^,^^12^

In this article, we use the term digital self-diagnosis tools to refer to the broad group of tools that take symptom information from nonclinical individuals ('patients'), process that information using digital technology, and produce diagnoses, treatment plans, or tailored healthcare advice. Within this broad category are variations in the mode of data input, the digital technologies used to process those data, and the form of diagnostic output, as illustrated in Figure 1.

**Figure 1:**
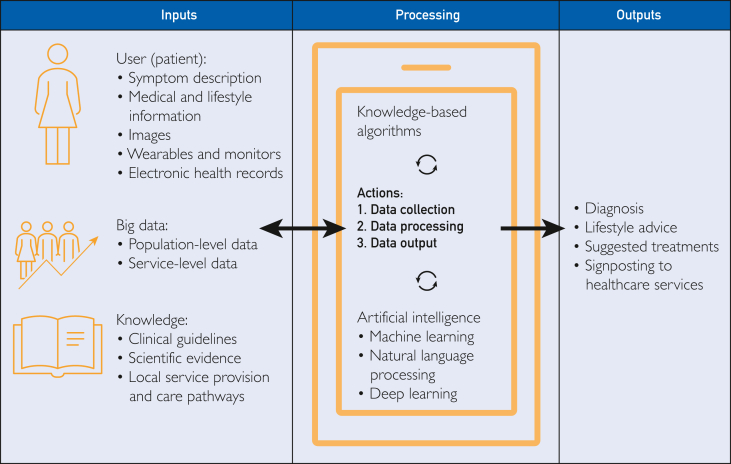
Schematic of digital self-diagnosis tool functions.

## Methods

**Figure 2:**
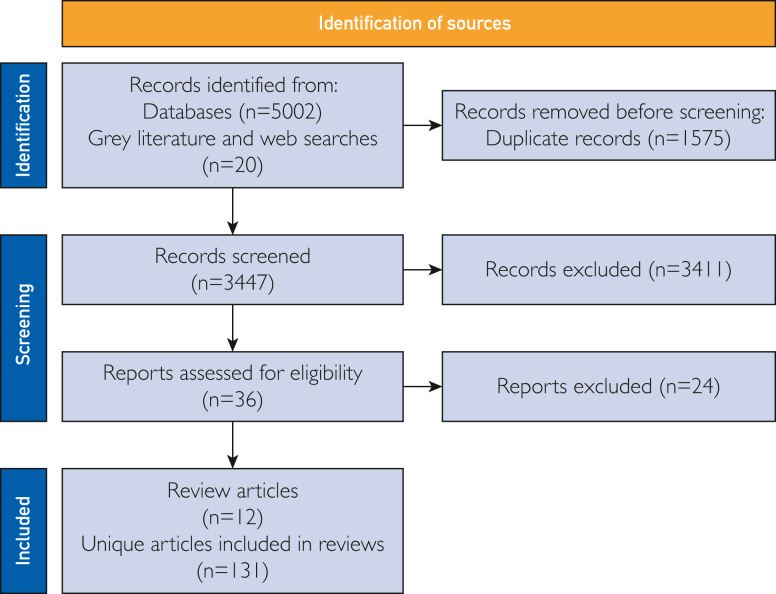
Flow diagram illustrating the process of article identification and screening.^13^

We conducted a narrative overview of reviews aiming to identify interdisciplinary insights from existing evidence syntheses on digital self-diagnosis tools that use AI, adhering to PRIOR guidelines for conducting and reporting on narrative reviews (Figure 2).^13^^,^^14^ Narrative reviews are suitable methodology for exploring and critically interpreting existing research findings and their broader implications.^15^ To support further research and discussion across disciplinary boundaries, we also sought to identify the terms used to describe digital self-diagnosis tools.

### Search Strategy and Selection Criteria

On 7 September 2023, we conducted a literature search in 3 bibliographic databases to identify relevant academic articles in the published literature: PubMed, Scopus, and Web of Science. These databases were selected because, together, they capture literature across many different disciplines relevant to healthcare. Google Scholar was also searched for grey literature, and reference sections of included articles were examined for additional relevant studies.

The search strategy used the following keywords selected to identify literature on self-diagnosis tools using AI: (*Artificial* AND *Self-diagnosis*); (*Artificial* AND *Intelligence* AND *Self* AND *Diagnosis*); (*Machine Learning* AND *Self-diagnosis*); and (*Machine* AND *Learning* AND *Self* AND *Diagnosis*). Using the scoping review by Aboueid et al^16^ as a starting point, we limited our search parameters to 2019-2023 to focus only on the newest reviews in the field. We included systematic reviews, meta-analyses, scoping reviews, narrative syntheses, and opinion pieces that discussed tools where users proactively entered personal health information to acquire a predicted diagnosis of their symptoms or triage advice.

Articles were excluded if they met any of the following criteria: (a) explored tools used by healthcare staff to facilitate diagnosis or support decision-making; (b) described only the computational mechanisms of a given tool; (c) described tools used by patients to monitor long-term health conditions; (d) described the use of AI by healthcare professionals (rather than users who are not healthcare professionals) in triage, neuroimaging, chronic disease management, or general health information; and (e) did not have an English language full text. We combined the results of the searches in EndNote 20 and removed duplicates. One author (A.M.) screened all articles using the title and abstract and then obtained full texts of potentially eligible articles. Three authors (A.M., A.R., E.C.) independently conducted the full text screening. Disagreement or uncertainty about inclusion was resolved by consensus discussion between all authors. Data extraction tables were used to collate bibliographic information and qualitative data (Supplemental Appendix 1, available online at https://www.mcpdigitalhealth.org/).

### Primary Analysis

All data extracted were reviewed by the research team and discussed collaboratively over a series of online meetings. Narrative review findings were initially drafted by the first author and supplemented with discipline-specific insights by the other authors.

### Secondary Analysis

When we started reading and analyzing the 12 reviews, we realized that the reviews had applied terminology in different ways, potentially influencing the original reviewers' interpretation of the included evidence and their overall findings. Clarity on language meaning and usage is essential for successful interdisciplinary inquiry.^17^ Therefore, we decided to extract data from studies reviewed in the 12 reviews. We summarized the analysis of the reviews and used specific articles in the reviews to highlight some key points that stood out in the analysis. This was particularly relevant for the section on terminology. Using a standard data extraction spreadsheet, we extracted from all articles included in the original reviews the terms used to describe digital self-diagnosis tool technologies and the definition, either given in the source text or, if undefined, as implied by usage (Supplemental Appendix 2, available online at https://www.mcpdigitalhealth.org/).

## Results

Database searches yielded 5022 articles. After deduplication, 3447 unique articles were screened. Title and abstract screening resulted in 36 articles being selected for full text screening. Of those articles, 12 evidence syntheses were included in this review (Figure 2^13^; Table 2^5^^,^^6^^,^^16^^,^^18^, ^19^, ^20^, ^21^, ^22^, ^23^, ^24^, ^25^, ^26^). We extracted qualitative data from these 12 syntheses and the 131 unique articles contained within them (limited data from title and abstract could only be extracted from the 5 foreign language articles, and 12 articles were not available in full text). Data were not extracted from the articles (n=83) included in the review of Char et al^18^ because we could not identify which articles in their analysis corresponded specifically to the use of digital self-diagnosis tools.

**Table 2:** Included Review Articles

Reference, year	Title
Aboueid et al,^16^ 2019	The use of artificially intelligent self-diagnosing digital platforms by the general public: scoping review
Chambers et al,^6^ 2019	Digital and online symptom checkers and health assessment/triage services for urgent health problems: systematic review
Char et al,^18^ 2020	Identifying ethical considerations for machine learning healthcare applications
Gottliebsen and Petersson,^19^ 2020	Limited evidence of benefits of patient operated intelligent primary care triage tools: findings of a literature review
Ilicki,^20^ 2022	Challenges in evaluating the accuracy of AI-containing digital triage systems: A systematic review
Jovicic,^21^ 2020	eHealth for risk screening and early diagnosis: a scoping review on the accuracy and availability of online diagnostic tools
Müller et al,^22^ 2022	Ethical, legal, and social aspects of symptom checker applications: a scoping review
Pairon et al,^23^ 2023	A scoping review on the use and usefulness of online symptom checkers and triage systems: how to proceed?
Radionova et al,^24^ 2023	Impacts of symptom checkers for laypersons’ self-diagnosis on physicians in primary care: scoping review
Riboli-Sasco et al,^5^ 2023	Triage and diagnostic accuracy of online symptom checkers: systematic review
Wallace et al,^25^ 2022	The diagnostic and triage accuracy of digital and online symptom checker tools: a systematic review
You et al,^26^ 2022	User experience of symptom checkers: a systematic review

### Characteristics of the Included Reviews and Articles

There were an equal number of traditional systematic reviews (n=5) and scoping reviews (n=5), with a minority of narrative literature reviews (n=2). Most (n=9, 75%) came from a health-related discipline (determined by first author affiliation), such as medicine, public health, and health sciences.^5^^,^^6^^,^^16^^,^^18^, ^19^, ^20^, ^21^, ^22^, ^23^, ^24^, ^25^ One review was from the social sciences (medical humanities),^22^ whereas 2 were from the bioinformatics field.^20^^,^^26^ Most (n=7) considered empirical research and previous evidence syntheses.

Most articles included in the reviews were empirical studies (71%). All articles included in the reviews were published between 2001 and 2022 in disciplines including medicine (57%), health sciences (14%), computer science/bioinformatics (12%), and social sciences (13%).

### Narrative Review

The following section summarizes the key themes and issues identified by the included reviews.

#### Development

Most of the included reviews explored the development of digital self-diagnosis tools. Related themes included transparency and the heterogeneity of available AI-centered self-diagnosis tools.

Authors expressed concerns about a lack of transparency in tool development.^24^^,^^25^ Because many tools are developed by private companies, it is often unclear how the algorithms powering them have been designed and trained.^24^^,^^25^

Further questions arise around the origins of the datasets used to train these tools. Imbalances and underrepresentation of demographic characteristics and disease presentations in datasets can introduce bias into tool outputs and reduce generalizability.^18^^,^^25^

The landscape of digital self-diagnosis tool development is characterized by heterogeneity and inconsistency. Many tools using diverse technologies have been developed, often lacking external validation, a problem exacerbated by the aforementioned lack of transparency.^5^^,^^20^^,^^25^ Furthermore, some tools are designed to cover many medical conditions, whereas others are more specialized, challenging meaningful comparisons of tool performance and potential application.^16^^,^^22^

#### Implementation

Several aspects of implementing digital self-diagnosis tools are described in the reviews, including user characteristics and experience and real-world applications.

Research on user demographic characteristics suggests that digital self-diagnosis tools have a diverse user base, reflecting the popularity of these tools with the general population and potentially reflecting the embeddedness of digital technologies in everyday life.^6^^,^^23^ However, existing empirical work shows that users are more likely to be young, female, and currently employed.^6^^,^^26^ Although there is some evidence that users tend to be highly educated,^23^ You et al^26^ found that users tended to have low health literacy and high technology literacy.

Overall, the literature reviewed shows that users of digital self-diagnosis tools are satisfied and have positive experiences, finding the tools valuable and usable.^6^^,^^23^ Problems with medical jargon have also been reported, with lay audiences encountering challenges in interpreting health-related information.^23^^,^^24^ There is currently limited empirical research on the transferability of digital self-diagnosis tools to real-world contexts, with most published studies presenting findings from simulations or controlled experiments.^23^^,^^25^^,^^26^

#### Impact

All included reviews reported expected impacts of digital self-diagnosis tools. Relevant themes included accuracy, user compliance, patient/physician interactions, and implications for healthcare systems.

All included reviews mentioned 2 aspects of diagnostic tool accuracy: diagnostic accuracy and triage accuracy. Diagnostic accuracy correctly discriminates between a target health condition (disease) and health. In contrast, triage accuracy refers to the ability to assign urgency to enable appropriate care.^23^ The current evidence base on diagnostic and triage accuracy of digital self-diagnosis tools in use is limited. Given this lack of evidence on accuracy, some authors have asked whether the implementation of digital self-diagnosis tools will ultimately improve users’ health.^16^

Digital self-diagnosis tools tend to be developed with a risk-averse triage disposition, meaning that most users will be advised to seek medical attention, even when it may not be necessary, potentially increasing healthcare demand.^5^^,^^6^^,^^19^^,^^23^ Although more balanced algorithms might reduce unnecessary demand, risk-aversion is a safety feature to avoid underdiagnosing clinically significant conditions that require medical intervention.^23^ Instances of under-triage (ie, underestimating the severity of symptoms) could result in delayed care, adverse health outcomes, and uncertain legal liabilities for the different parties involved in tool development.^5^

Even if a self-diagnosis tool claims to achieve near-perfect accuracy, social factors influencing how users interact with these tools must be considered.^20^ It has been claimed that self-diagnosis tools will empower patients by providing information and giving them greater control over their own health.^23^^,^^24^ However, it is suggested that, although users appear satisfied using these tools, they do not often follow the recommended action.^24^ Authors have raised the issue of exploring how users interpret and use healthcare advice from self-diagnosis tools as essential to understanding care-seeking behaviors.^19^^,^^21^

Several reviews also called for further research on how self-diagnosis tools can efficiently deliver intended health outcomes, arguing that the increasing complexity of modern healthcare systems necessitates their development.^20^^,^^23^^,^^25^ However, Radionova et al^24^ noted a lack of research on the impact of digital self-diagnosis tools on aspects of patient–physician relationship, including shared decision-making and patient autonomy. Likewise, although AI-based self-diagnosis tools could impact healthcare processes, workloads, and access, the evidence for this is limited and inconclusive.^18^^,^^19^

#### Oversight

Most of the reviews explored oversight of digital self-diagnosis tools, including the need for regulation, data privacy and ethical concerns, and conflicts of interest. However, the existing evidence base is limited, and discussion is mainly speculative, with legal literature on self-diagnosis tools noted to be scarce.^22^

Researchers suggested the establishment of regulatory practice standards and evaluation guidelines, similar to other medical products, should be a priority.^19^^,^^20^ The included reviews have also identified specific regulatory gaps around privacy and data protection, reflecting concerns about data ownership and traceability of sensitive individual user medical information once entered into a tool.^18^^,^^22^ Authors suggested future legal considerations should include patient safety, data security, and liability.^16^^,^^18^ Additionally, Wallace et al^25^ highlighted a need for greater regulatory scrutiny and postmarket surveillance of AI-based self-diagnosis tools.

Ethical perspectives on digital self-diagnosis tools raised in the reviews were complex and cross-disciplinary; these included monetizing medical data, techno-optimism, and premature adoption of immature technologies.^19^^,^^22^ According to the existing literature, discussions about ethical implications should focus on all stages of development, implementation, and predicted impact of AI-centered self-diagnosis tools, aiming to increase transparency, informed decision-making, and better understanding of the tools’ limitations.^18^^,^^22^ Authors advocated for increased cross-disciplinary collaboration to enable further critical discussions and identification of ethical issues.^18^^,^^22^

Finally, several included reviews raised concerns over potential conflicts of interest.^6^^,^^18^, ^19^, ^20^ Private companies that have developed self-diagnosis tools will have commercial and operational interests that conflict with users’ interests.^18^^,^^19^ Making inferences about a tool;s performance from nonindependent evaluations may be challenging.^6^^,^^19^ The reviews suggest that independent research and evaluative autonomy should address conflicts of interest by involving the disclosure of tool architecture and in-built values to allow for informed decision making.^18^^,^^20^

### Terminology

Each of the included reviews used a different term to describe digital health tools. Some specified user, purpose, technology, or platform, such as self-diagnosing digital platforms,^16^ algorithm-based self-diagnosis tools,^24^ AI-containing digital patient-facing triage tools,^20^ and patient operated intelligent primary care triage tools.^19^ Others favored more generic terms, such as symptom checkers,^5^ online health assessment or triage services,^6^ risk calculators,^21^ and machine learning healthcare applications,^18^ and required close reading to ascertain that they included discussion of digital self-diagnosis tools.

**Figure 3:**
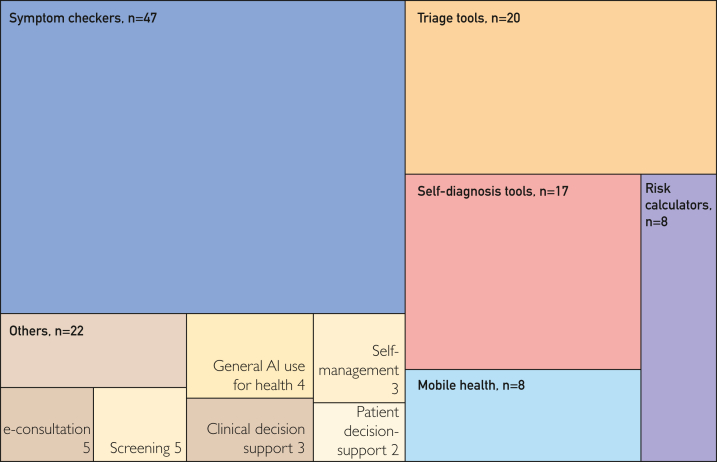
Primary terms used to describe the digital health tools discussed in review source articles.

An even greater diversity of terms was used to describe digital health tools in the review sources (Figure 3). The most common term used was symptom checkers (n=85), which referred to digital tools that require laypersons to input their symptoms to produce a set of likely diagnoses or healthcare triage advice. Terms often appeared with additional qualifications to denote specific technologies or tools (eg, AI-assisted symptom checkers and NHS111 Online). Of note, 8 reviews included source articles that primarily discussed digital health tools not for lay use (eg, CDS, screening, or risk assessment tools) or were designed to support remote healthcare consultations (eg, e-consultations).^6^^,^^16^^,^^19^^,^^21^^,^^22^^,^^24^, ^25^, ^26^ Further, 67 of the included source articles did not specifically mention AI.

It was frequently unclear which technological approach was used in a tool. It was possible to be confident that an article discussed an AI-based tool in only 34 (26%) source articles. Of these, 7 specified the AI approaches, including machine learning and natural language processing. There was frequently a lack of specificity on where the AI approach was deployed in a tool. For instance, a self-diagnosis tool labeled as AI could use a natural language processing chatbot interface to collect data that are then fed into a knowledge-based branching algorithm; this would be quite different from a tool using machine learning model to generate probable diagnoses from multiple types of self-reported and healthcare data.

In total, 84 articles referred only to tools using an algorithm, with no additional detail as to the nature of the algorithms and whether they used machine learning or other inference approaches beyond conventional software programming. Indeed, when its source articles were examined, a scoping review of artificially intelligent self-diagnosing digital platforms included articles describing digital tools that used knowledge-based branching algorithms without specification of them including AI technology.^16^

Only 2 of the included reviews directly addressed the heterogeneity of terminology used in the field.^21^^,^^24^ Radinova et al^24^ suggest that medical terminology (eg, online triage services) may overstate a tool’s ability to deliver clinically relevant decisions. At the same time, symptom checkers may convey a more advisory function.^24^ Ilicki^20^ specifically drew attention to the ambiguity of the term AI when applied to digital tools without further detail about what AI technique is being used and for what function.

## Discussion

Our review identified 12 evidence syntheses on digital self-diagnosis tools. Our analysis revealed critical gaps in their development, implementation, impact, and oversight, suggesting areas for future research. The authorship and publishing journals of these reviews reflect a health sciences focus on the topic with limited interdisciplinary perspectives. This section follows a discussion of the themes and gaps in the included reviews, taking an interdisciplinary viewpoint.

### Development

Artificial intelligence tools are only as good as the data used to train them. Where self-diagnosis tools are developed with training data that are unrepresentative of the intended population of tool users, there is a risk of biased outputs causing harm or delaying the appropriate seeking of medical attention, particularly affecting underrepresented groups, for example, women, ethnic minorities, and disadvantaged socioeconomic groups. For example, a scoping review of machine learning designed to classify potentially malignant skin cancers found that light skin types dominated training data sets and that the models were likely to underperform in patients with darker skin types.^27^

To our knowledge, there have not yet been any legal cases of medical negligence or personal injury due to bias in digital self-diagnosis tools, although there are judicial decisions relating to other legal frameworks that could be used as claims or arguments to enhance medical negligence actions involving digital self-diagnosis tools. In criminal law, an algorithm became the subject of intense scrutiny in a US court case (State of Wisconsin vs Eric L. Loomis). In the same year an investigation by ProPublica, a nonprofit investigative journalism organization, claimed that the proprietary COMPAS (Correctional Offender Management Profiling for Alternative Sanctions) algorithm exhibited gender and racial bias and unfairly influenced criminal court sentencing decisions.^28^

There is also a risk that capitalist profit motives may override the interests of users and the subjects of the big data being used to train the underlying models. This conflict is particularly concerning for the Global South, where privacy governance frameworks are less developed and/or enforced and the exploitative and extractive use of private data for training has been termed data colonialism.^29^^,^^30^

Developing standards for these tools will be necessary to ensure they are safe, effective, and trustworthy. Indeed, one of the objectives of the United Nations Global Initiative on AI for Health,^31^ launched in July 2023, is the development of technical standards for AI health solutions. In addition to standards for tools themselves, it has been suggested that AI development using healthcare and other public sector data should undergo an algorithm impact assessment audit to systematically evaluate potential effects on individuals, groups, and society.^32^

### Implementation

Trust will be critical to the successful use of digital self-diagnosis tools. Von Eschenbach^33^ proposed that successful implementation of AI would require developers and policymakers to build trust in the whole sociotechnical system, including why and how tools have been developed.

If self-diagnosis tools are to be deployed as part of a national healthcare system, it will be vital to assess the effect of socioeconomic exclusion on uptake. Expected reductions in healthcare demand may not be achieved if the end users cannot access self-diagnosis tools. Research into the use of digital technologies related to UK health outcomes has described a digital divide arising from differences in access to technology (affordability and infrastructure), digital literacy, experience, and trust.^34^ Similarly, studies on COVID-19–tracing applications found they were often met with mistrust and misunderstanding, particularly among marginalized and disadvantaged communities.^35^

### Impact

Digital self-diagnosis tools have the potential to impact not just their users but also users’ relationships with clinicians, the healthcare system, and broader society. Most assessments of self-diagnosis tools have focused on the accuracy of diagnosis, as measured by comparing tool outputs with a gold standard, often a diagnosis made by an expert clinician. However, this one-dimensional view ignores the interpersonal, emotional, and communicative aspects of healthcare.^36^^,^^37^ Understanding the inherent uncertainties of medical practice and recognizing the blind spots created when encoding human experience as data will be essential if self-diagnosis tools are to be a valuable addition to modern healthcare.^38^

The entry of commercial technology products into spaces traditionally served by public sector institutions can have unanticipated social implications. As the case of Babylon Health’s much-hyped GP At Hand self-help tool demonstrated, the appearance of commercial services and products renders the healthcare space a market that private actors will attempt to dominate but can leave if expected profits do not transpire.^39^ The exit of Babylon Health from the UK caused considerable disruption to local NHS services, highlighting the vulnerability of the social contract between citizens and public institutions when profit enters the picture.^40^^,^^41^

### Oversight

The regulatory landscape for digital self-diagnosis tools is complex and evolving. Tools offering definitive diagnoses or specific treatments are typically classified as medical devices, subject to risk-based regulation. Consumer protection laws often apply to exempt tools.^42^ Artificial intelligence challenges traditional hardware-based regulations, leading to new classifications like Software as a Medical Device (SaMD) and AI as a Medical Device (AIaMD).^43^ Most digital self-diagnosis tools, intended for medical purposes without being part of a hardware medical device, would be classified as SaMD. Proposed SaMD frameworks include transparency requirements, real-world performance monitoring, and adaptive algorithms.^42^^,^^44^

As nations compete in AI development, they also race to establish facilitating legislation.^45^ The Council of Europe’s Framework Convention on Artificial Intelligence (May 2024), the first international AI treaty, promotes innovation while safeguarding human rights, democracy, and rule of law. Its intersection with existing digital health legislation remains unclear, and not all jurisdictions will sign.^46^ International agreements notwithstanding, governments can be expected to continue to compete to attract AI investment by targeted changes to their laws in fields as diverse as taxation, data protection, intellectual property, procurement, and tort law.^45^

The liability for harm resulting from self-diagnosis algorithms remains unclear. Potential legal remedies span international conventions, local civil and criminal statutes, and case law. Categorizing these tools within appropriate legal frameworks and defining liable parties will be challenging.

### Terminology

The variety of applicable terminology, although indicative of an emerging field of study, obstructs crossdisciplinary inquiry. The term *AI*, without qualifiers as to the specific technology, is being used to describe technologies ranging from simple branching logic algorithms to complex deep neural networks. Thus, the term *AI* hides a considerable variety of computational approaches, each with different potential concerns and impacts. Exaggerated claims of *AI-powered* tools have the potential to both misleadingly overhype and deter people from engaging with them owing to unfounded fears.

### Limitations

The preponderance of terms describing self-diagnosis tools and related technologies means that any review risks missing discussions in academic fields using atypical terminology. For resource reasons, we limited the review to available English language sources only. Moreover, review-type articles are not typical in all fields; they are rare in some humanities and social science disciplines, such as law. Our review may, therefore, have missed some relevant perspectives.

### Future Research

Interdisciplinary research collaboration could open further insightful avenues and foster a more informed public discourse, promoting responsible development and implementation. The political economies that underpin digital self-diagnosis tools are still to be explored, such as the role of large technological companies and market competition dynamics, the impact of future regulation, integration with existing healthcare systems, and how consumers interact with tools. Existing reviews have touched on cultural imaginaries, such as the empowered patient and technology as savior; however, this topic deserves interpretation within other cultural narratives, including the quantified-self movement and arguments around surveillance and control. Fundamentally, there is a need for ethical deliberation to go beyond instrumentalization toward considering the more profound moral questions around their impact on the nature of health and disease, the doctor–patient relationship, autonomy, and equity, with particular attention to exploitation of data and planetary resources.^47^^,^^48^

## Conclusion

This review synthesizes key themes from the literature on digital self-diagnosis tools, adopting an interdisciplinary approach. We identified gaps and critical areas for future research across all stages of these tools’ lifecycles. The diverse challenges uncovered highlight the necessity for multiagency and multidisciplinary efforts.

## Potential Competing Interests

Dr Carvalho was supported by University of Dundee, Institute for Social Sciences (ISSR) Interdisciplinary Incubator Grant (IIG) and Wellcome Trust Block grant awarded to University of Dundee. Prof Daly reports institutional grants from Economic and Social Research Council; Royal Society of Edinburgh, European Commission, UKRI, Scottish Universities Insight Institute, Norwegian Research Council, British Telecom, and Leverhulme Trust and travel support from UK Embassy in Manila, Philippines, and University of Newcastle Singapore. Ms Malone reports institutional grants from Economic and Social Research Council (ESRC) and UKRI, an Early Career Fellowship from Scottish Universities Law Institute and travel and scholarship support from University of Dundee Staff development fund. Dr Kerasidou was supported by The Wellcome Trust (grant no. 203132/Z/16/Z, at the time of submission of this article and for the 4 months before submission). The other authors report no competing interests.
