# Supplementary material for: Artificial Intelligence in Digital Self-Diagnosis Tools: A Narrative Overview of Reviews
Source: Mayo Clin Proc Digit Health. 2025 Jun 10;3(3):100242. doi: 10.1016/j.mcpdig.2025.100242 (PMC12271431; doi:10.1016/j.mcpdig.2025.100242)
Supplement: Supplementary Appendix 1 [file mmc1.pdf]

Review	Title
Pairon et al., 2023	A scoping review on the use and usefulness of online symptom checkers and triage systems: How to proceed?

Chambers et al (2019)	Digital and online symptom checkers and health assessment/triage services for urgent health problems: systematic review

Aboueid et al (2019)	The Use of Artificially Intelligent Self-Diagnosing Digital P

Wallace et al., 2022 The diagnostic and triage accuracy of digital and online s

Gottliebsen & Petersson, 2020	Limited evidence of benefits of patient operated intelligent primary care triage tools: findings of a literature review

Jovicic, 2020	eHealth for risk screening and early diagnosis: A scoping review<br>on the accuracy and availability of online diagnostic tools

Muller et al., 2022	Ethical, legal, and social aspects of symptom checker applications:<br>a scoping review

Radionova et al., 2023	Impacts of Symptom Checkers for Laypersons' Self-diagnosis on Physicians in Primary Care: Scoping Review
You et al., 2022	User Experience of Symptom Checkers: A Systematic Review

Riboli-Sasco et al., 2023	Triage and Diagnostic Accuracy of Online Symptom Checkers:<br>Systematic Review
Char et al., 2020	Identifying Ethical Considerations for Machine Learning Healthcare Applications

Abstract	1st author's discipline
Background: Patients are increasingly turning to the Internet for health information. Numerous online symptom checkers and digital triage tools are currently available to the general public in an effort to meet this need, simultaneously acting as a demand management strategy to aid the overburdened health care system. The implementation of these services requires an evidence-based approach, warranting a review of the available literature on this rapidly evolving topic.<br><br>Objective: This scoping review aims to provide an overview of the current state of the art and identify research gaps through an analysis of the strengths and weaknesses of the presently available literature.<br><br>Methods: A systematic search strategy was formed and applied to six databases: Cochrane library, NICE, DARE, NIHR, Pubmed, and Web of Science. Data extraction was performed by two researchers according to a pre-established data charting methodology allowing for a thematic analysis of the results.<br><br>Results: A total of 10,250 articles were identified, and 28 publications were found eligible for inclusion. Users of these tools are often younger, female, more highly educated and technologically literate, potentially impacting digital divide and health equity. Triage algorithms remain risk-averse, which causes challenges for their accuracy. Recent evolutions in algorithms have varying degrees of success. Results on impact are highly variable, with potential effects on demand, accessibility of care, health literacy and syndromic	medicine

Objectives In England, the NHS111 service provides assessment and triage by telephone for urgent health problems. A digital version of this service has recently been introduced. We aimed to systematically review the evidence on digital and online symptom checkers and similar services.

Design Systematic review.

Data sources We searched Medline, Embase, the Cochrane Library, Cumulative Index to Nursing and Allied Health Literature (CINAHL), Health Management Information Consortium, Web of Science and ACM Digital Library up to April 2018, supplemented by phrase searches for known symptom checkers and citation searching of key studies.

Eligibility criteria Studies of any design that evaluated a digital or online symptom checker or health assessment service for people seeking advice about an urgent health problem.

Data extraction and synthesis Data extraction and quality assessment (using the Cochrane Collaboration version of QUADAS for diagnostic accuracy studies and the National Heart, Lung and Blood Institute tool for observational studies) were done by one reviewer with a sample checked for accuracy and consistency. We performed a narrative synthesis of the included studies structured around pre-defined research questions and key outcomes.

Results We included 29 publications (27 studies). Evidence on patient

health sciences

Background:

health sciences

Self-diagnosis is the process of diagnosing or identifying a medical condition in oneself. Artificially intelligent digital platforms for self-diagnosis are becoming widely available and are used by the general public; however, little is known about the body of knowledge surrounding this technology.

Objective:

The objectives of this scoping review were to (1) systematically map the extent and nature of the literature and topic areas pertaining to digital platforms that use computerized algorithms to provide users with a list of potential diagnoses and (2) identify key knowledge gaps.

Methods:

The following databases were searched: PubMed (Medline), Scopus, Association for Computing Machinery Digital Library, Institute of Electrical and Electronics Engineers, Google Scholar, Open Grey, and ProQuest Dissertations and Theses. The search strategy was developed and refined with the assistance of a librarian and consisted of 3 main concepts: (1) self-diagnosis; (2) digital platforms; and (3) public or patients. The search generated 2536 articles from which 217 were duplicates. Following the Tricco et al 2018 checklist, 2 researchers screened the titles and abstracts (n=2316) and full texts (n=104), independently. A total of 19 articles were included for review, and data were retrieved following a data-charting form that was pretested by the research team.

Results:

Digital and online symptom checkers are an increasingly adopted class of health technologies that enable patients to input their symptoms and biodata to produce a set of likely diagnoses and associated triage advice. However, concerns regarding the accuracy and safety of these symptom checkers have been raised. This systematic review evaluates the accuracy of symptom checkers in providing diagnoses and appropriate triage advice. MEDLINE and Web of Science were searched for studies that used either real or simulated patients to evaluate online or digital symptom checkers. The primary outcomes were the diagnostic and triage accuracy of the symptom checkers. The QUADAS-2 tool was used to assess study quality. Of the 177 studies retrieved, 10 studies met the inclusion criteria. Researchers evaluated the accuracy of symptom checkers using a variety of medical conditions, including ophthalmological conditions, inflammatory arthritides and HIV. A total of 50% of the studies recruited real patients, while the remainder used simulated cases. The diagnostic accuracy of the primary diagnosis was low across included studies (range: 19–37.9%) and varied between individual symptom checkers, despite consistent symptom data input. Triage accuracy (range: 48.8–90.1%) was typically higher than diagnostic accuracy. Overall, the diagnostic and triage accuracy of symptom checkers are variable and of low accuracy. Given the increasing push towards adopting this class of technologies across numerous health systems, this study demonstrates that reliance upon symptom checkers could pose significant patient safety hazards. Large-scale primary studies, based upon real-world data, are warranted to demonstrate the adequate performance of these technologies in a manner that is non-inferior to current best

medicine

There is consistent evidence that the workload in general practices is substantially increasing. The digitalisation of healthcare including the use of artificial intelligence has been suggested as a solution to this problem. We wanted to explore the features of intelligent online triage tools in primary care by conducting a literature review.

no mention

Method

A systematic literature search strategy was formulated and conducted in the PubMed database and the Cochrane Library. Articles were selected according to inclusion/exclusion criteria. Results and data were systematically extracted and thematically analysed. 17 articles of that reported large multimethod studies or smaller diagnostic accuracy tests on clinical vignettes were included. Reviews and expert opinions were also considered.

Results

There was limited evidence on the actual effects and performance of triage tools in primary care. Several aspects can guide further development: concepts of system design, system implementation and diagnostic performance. The most important findings were: a need to formulate evaluation guidelines and regulations; their assumed potential has not yet been met; a risk of increased or redistribution of workloads and the available symptom checker systems seem overly risk averse and should be tested in real-life settings.

Conclusion

This review identified several features associated with the design and

Introduction

Patient-operated digital triage systems with AI components are becoming increasingly common. However, previous reviews have found a limited amount of research on such systems' accuracy. This systematic review of the literature aimed to identify the main challenges in determining the accuracy of patient-operated digital AI-based triage systems.

Methods

A systematic review was designed and conducted in accordance with PRISMA guidelines in October 2021 using PubMed, Scopus and Web of Science. Articles were included if they assessed the accuracy of a patient-operated digital triage system that had an AI-component and could triage a general primary care population. Limitations and other pertinent data were extracted, synthesized and analysed. Risk of bias was not analysed as this review studied the included articles' limitations (rather than results). Results were synthesized qualitatively using a thematic analysis.

Results

The search generated 76 articles and following exclusion 8 articles (6 primary articles and 2 reviews) were included in the analysis. Articles' limitations were synthesized into three groups: epistemological, ontological and methodological limitations. Limitations varied with regards to intractability and the level to which they can be addressed through methodological choices. Certain methodological limitations related to testing triage systems using vignettes can be addressed through methodological adjustments, whereas epistemological and

Introduction: Health applications under the form of symptom checkers or other diagnostic tools have promising implications to not only reduce some of the burdens of the modern health care market, but also in empowering its users in becoming increasingly and more positively involved in their own health care. However, not much is known about the availability of such tools, their diagnostic capabilities and accuracy, and their impact on the behaviors and attitudes of its users.

Objective: To conduct a scoping review in order to explore these gaps and to report what is actually known about diagnostic tools across the literature by mapping all known tools and studies in that subject.

Methods: A search strategy was devised, and three databases were searched for potential studies: PubMed, Scopus, and PsycInfo. 330 studies were identified and were subject to full-text reviews. We included studies of any design as long as they appraised the accuracy of diagnostic tools available to the general public and provided information on the behavioral impact upon the use of such. Thus, 31 studies were selected for final review. Data was extracted in tables, where the characteristics of the tools and studies were summarized and presented.

Results: Three different types of diagnostic tools have been

Symptom Checker Applications (SCA) are mobile applications often designed for the end-user to assist with symptom assessment and self-triage. SCA are meant to provide the user with easily accessible information about their own health conditions. However, SCA raise questions regarding ethical, legal, and social aspects (ELSA), for example, regarding fair access to this new technology. The aim of this scoping review is to identify the ELSA of SCA in the scientific literature. A scoping review was conducted to identify the ELSA of SCA. Ten databases (e.g., Web of Science and PubMed) were used. Studies on SCA that address ELSA, written in English or German, were included in the review. The ELSA of SCA were extracted and synthesized using qualitative content analysis. A total of 25,061 references were identified, of which 39 were included in the analysis. The identified aspects were allotted to three main categories: (1) Technology; (2) Individual Level; and (3) Healthcare system. The results show that there are controversial debates in the literature on the ethical and social challenges of SCA usage. Furthermore, the debates are characterised by a lack of a specific legal perspective and empirical data. The review provides an overview on the spectrum of ELSA regarding SCA. It offers guidance to stakeholders in the healthcare system, for example, patients, healthcare professionals, and insurance providers and could be used in future empirical research to investigate the perspectives of those affected, such as users.

Background:
Symptom checkers (SCs) for laypersons' self-assessment and preliminary self-diagnosis are widely used by the public. Little is known about the impact of these tools on health care professionals (HCPs) in primary care and their work. This is relevant to understanding how technological changes might affect the working world and how this is linked to work-related psychosocial demands and resources for HCPs.

Objective:
This scoping review aimed to systematically explore the existing publications on the impacts of SCs on HCPs in primary care and to identify knowledge gaps.

Methods:
We used the Arksey and O'Malley framework. We based our search string on the participant, concept, and context scheme and searched PubMed (MEDLINE) and CINAHL in January and June 2021. We performed a reference search in August 2021 and a manual search in November 2021. We included publications of peer-reviewed journals that focused on artificial intelligence- or algorithm-based self-diagnosing apps and tools for laypersons and had primary care or nonclinical settings as a relevant context. The characteristics of these studies were described numerically. We used thematic analysis to identify core themes. We followed the PRISMA-ScR (Preferred Reporting Items for Systematic Reviews and Meta-Analyses extension for Scoping Reviews) checklist to report the study.

This review reports the user experience of symptom checkers, aiming to characterize users studied in the existing literature, identify the aspects of user experience of symptom checkers that have been studied, and offer design suggestions. Our literature search resulted in 31 publications. We found that (1) most symptom checker users are relatively young;(2) eight relevant aspects of user experience have been explored, including motivation, trust, acceptability, satisfaction, accuracy, usability, safety/security, and functionality;(3) future symptom checkers should improve their accuracy, safety, and usability. Although many facets of user experience have been explored, methodological challenges exist and some important aspects of user experience remain understudied. Further research should be conducted to explore users' needs and the context of use. More qualitative and mixed-method studies are needed to understand actual users' experiences in the future.

Background:
In the context of a deepening global shortage of health workers and, in particular, the COVID-19 pandemic, there is growing international interest in, and use of, online symptom checkers (OSCs). However, the evidence surrounding the triage and diagnostic accuracy of these tools remains inconclusive.

Objective:
This systematic review aimed to summarize the existing peer-reviewed literature evaluating the triage accuracy (directing users to appropriate services based on their presenting symptoms) and diagnostic accuracy of OSCs aimed at lay users for general health concerns.

Methods:
Searches were conducted in MEDLINE, Embase, CINAHL, Health Management Information Consortium (HMIC), and Web of Science, as well as the citations of the studies selected for full-text screening. We included peer-reviewed studies published in English between January 1, 2010, and February 16, 2022, with a controlled and quantitative assessment of either or both triage and diagnostic accuracy of OSCs directed at lay users. We excluded tools supporting health care professionals, as well as disease- or specialty-specific OSCs. Screening and data extraction were carried out independently by 2 reviewers for each study. We performed a descriptive narrative synthesis.

Results:
Along with potential benefits to healthcare delivery, machine learning healthcare applications
(ML-HCAs) raise a number of ethical concerns. Ethical evaluations of ML-HCAs will need to
structure the overall problem of evaluating these technologies, especially for a diverse group
of stakeholders. This paper outlines a systematic approach to identifying ML-HCA ethical concerns, starting with a conceptual model of the pipeline of the conception, development, implementation of ML-HCAs, and the parallel pipeline of evaluation and oversight tasks at
each stage. Over this model, we layer key questions that raise value-based issues, along with
ethical considerations identified in large part by a literature review, but also identifying some
ethical considerations that have yet to receive attention. This pipeline model framework will
be useful for systematic ethical appraisals of ML-HCA from development through implementation, and for interdisciplinary collaboration of diverse stakeholders that will be required to understand and subsequently manage the ethical implications of ML-HCAs.

journal	type
Frontiers in Medicine	scoping review

terms used: tools	definition
online symptom checkers / self-triage tools	no mention

Digital and online symptom checkers and health assessment/triage services	Digital and online symptom checkers and assessment services are used by patients seeking guidance about health problems, including some that may require urgent action. These services generally provide people with possible alternative diagnoses based on their reported symptoms and/or suggest a course of action (eg, self-care, make a general practitioner (GP) appointment or go to an emergency department.

Artificially Intelligent Self-Diagnosing Digital Platforms	Notably, AI has become incorporated in computerized diagnostic decision support systems, which were initially developed for health professionals. These platforms have now become readily available to the general public and are known as self-diagnosing apps or symptom checkers, which include the Mayo Clinic symptom checker, Babylon Health, the Ada health app, and the K Health app. On the basis of the medical information and symptoms provided by an individual, these digital platforms perform 2 main functions: (1) provide individuals with a list of potential diagnoses and (2) assist with triage. In this review, self-diagnosing digital platforms were defined as platforms that utilize algorithms to provide a list of potential diagnoses to the user based on the medical information and symptoms provided.

digital and online symptom checker tools	Digital and online symptom checkers are application or software tools that enable patients to input their symptoms and biodata to produce a set of differential diagnoses and clinical triage advice. The diagnostic function of symptom checkers is to provide a list of differential diagnoses, ranked by likelihood1. The triage function highlights to end-users the most appropriate course of action regarding their potential diagnosis, which typically includes seeking urgent care; contacting their general practitioner; or self-care.

patient<br>operated intelligent primary care triage<br>tools	digital tools that<br>can be accessed online and operated by the<br>patient. Furthermore, the main component<br>of the tools was to be triaging to enhance<br>the patient’s decision on choosing between<br>such outcomes as self-management, seeking<br>acute medical attention or seeking a planned<br>general practitioner (GP) consultation. In<br>addition, the digital tools were to feature at<br>least some level of AI, defined as the theory<br>advice and using a digital triage tool with some<br>level of AI<br>involvement. The triage tools were to be<br>patient operated.

N of unique terms: tools	terms used: AI
2	algorithms

3 no mention

1 algorithms

1 probably algorithms (not clear)

1 AI

definition	terms used: AI variable	notes
no mention	no mention	

no mention	no mention

no mention	no mention

no mention (they just mention in the conclusion, as a recommendation, that 'several studies have suggested that symptom checkers performance may improve over time given increased exposure to data upon which an AI-centred model may iterate. This is especially pertinent as adaptive AI algorithms (where there is continuous learning from new data and subsequent algorithm modification) are incorporated into symptom checkers.'	no mention

no mention	no mention
