# Supplementary material for: Artificial Intelligence in Digital Self-Diagnosis Tools: A Narrative Overview of Reviews
Source: Mayo Clin Proc Digit Health. 2025 Jun 10;3(3):100242. doi: 10.1016/j.mcpdig.2025.100242 (PMC12271431; doi:10.1016/j.mcpdig.2025.100242)
Supplement: Supplementary Appendix 2 [file mmc2.pdf]

authors (date)	1st author's discipline
Schmieding et al (2022)	medical informatics
Kujala et al (2022)	computer science
Tsai et al., (2022)	information science
Dickson et al (2022)	medicine
Chan et al (2021)	medicine
Turner et al (2021)	health sciences
Schmieding et al (2021)	medical informatics
Yu et al (2021)	medicine
You & Gui (2021)	health informatics
Aboueid et al (2021)	public health
Cross et al (2021)	No mention
Morse et al (2020)	medicine
Schmieding et al (2020)	medical informatics
Hill et al (2020)	No mention
Gottliebsen et al (2020)	medicine
Sutham et al (2020)	medicine
Donovan et al (2020)	medicine
Meyer et al (2020)	medicine
Chambers et al (2019)	health sciences
Aboueid et al (2019)	health sciences
Millenson et al (2018)	medicine
Verzantvoort et al (2018)	health sciences
Polynskaya et al (2018)	No mention
Giesen et al (2017)	health sciences
Elliot et al (2015)	public health
Semigran et al (2015)	medicine
Lupton et al (2015)	social sciences
Nijland et al (2010)	psychology
Babylon Health (2017)	no mention
Berry et al (2015)	No mention
Berry et al (2017)	No mention
Berry et al (2017)	No mention
Kellerman et al (2010)	medicine
Little et al (2017)	medicine
Luger et al (2014)	medicine
Marco-Luiz et al (2017)	medicine
Middleton et al (2016)	No mention
Nagykaldi et al (2010)	medicine
Nijland et al (2010)	psychology
Poote et al (2014)	psychology
Prince et al (2013)	medicine
Semigran et al (2016)	medicine
Semigran et al (2015)	medicine
Sole et al (2006)	health sciences
Yardley et al (2010)	psychology
Carter et al (2018)	medicine

Cowie et al (2018)	health sciences
Madan (2014)	No mention
NHS England (2017)	No mention
Nijland et al (2009)	psychology
Lanseng & Andreassen (2007)	marketing
Bisson et al (2014)	medicine
Bisson et al (2016)	medicine
Copeland et al (2018)	computer science
Farmer et al (2011)	medicine
Hagemen et al (2015)	medicine
Lanseng et al (2007)	marketing
Luger et al (2014)	medicine
Powley et al (2016)	medicine
Davies et al (2019)	medicine
Nurek et al (2015)	public health
Bauer et al (2017)	psychology
Weldegebrial et al (2016)	health informatics
Boulos et al (2014)	health sciences
Flaherty (2014)	law
Jutel & Lupton (2015)	nursing
Kao & Liebovitz (2017)	medicine
Lupton et al (2015)	social sciences
Morita et al (2017)	medicine
Ryan & Wilson (2008)	medicine
Semigran et al (2015)	medicine
Hagemen et al (2015)	medicine
Berry et al (2019)	medicine
Hill et al (2020)	No mention
Shen et al (2019)	medicine
Yu et al (2020)	medicine
Arancibia et al (2019)	medicine
Powley et al (2016)	medicine
Yoshida & Clark (2021)	dentistry
Gilbert et al (2020)	No mention
Fraser et al (2018)	biomedical informatics
Coiera (2018)	no mention
Razzaki et al (2018)	No mention
Ghosh et al (2018)	No mention
Millenson et al (2018)	medicine
Cowie et al (2018)	health sciences
Carter et al (2018)	medicine
Edwards et al (2017)	medicine
Casey et al (2017)	medicine
Spoelman et al (2016)	public health
Semigran et al (2015)	medicine
Semigran et al (2016)	medicine
Jutel & Lupton (2015)	nursing
Elwyn et al (2011)	public health

Nijland et al (2010)	psychology
Nijland et al (2009)	psychology
Sadeghi et al (2001)	no mention
Semigran et al (2015)	medicine
Gilbert et al (2020)	No mention
Gottliebsen & Petersson (2020)	medicine
Chambers et al (2019)	health sciences
Middleton et al (2016)	No mention
Ghosh et al (2018)	No mention
Razzaki et al (2018)	No mention
Entezarjou et al (2020	medicine
Berry et al (2019)	medicine
Bisson et al (2014)	medicine
Bisson et al (2016)	medicine
Davies et al (2019)	medicine
Donker et al (2009)	No mention
Farmer et al (2011)	medicine
Farvolden et al (2003)	psychology
Ferrero et al (2013)	medicine
Hagemen et al (2015)	medicine
Heikes et al (2007)	No mention
Kafle et al (2018)	No mention
Kim & Lee (2019)	computer science
Kwan et al (2012)	health sciences
Levy et al (2008)	public health
Lippi & Sanchis-Gomar (2018)	clinical biochemistry
Losina et al (2015)	medicine
Losina et al (2017)	medicine
Maier et al 2014)	medicine
Middleton et al (2016)	No mention
Nijland et al (2010)	psychology
Ngoo et al (2017)	medicine
Poote et al (2014)	psychology
Powley et al (2016)	medicine
Prince et al (2013)	medicine
Semigran et al (2015)	medicine
Ruotsalo & Lipsanen (2018)	computer science
Shen et al (2019)	medicine
Sole et al (2006)	health sciences
Fiske et al (2020)	ethics
Dunn (2020)	biomedical informatics
Fraser et al (2018)	biomedical informatics
Jutel & Lupton (2015)	nursing
Lupton et al (2015)	social sciences
Kramer (2017)	
Merz et al (2018)	
Powley et al (2016)	medicine

Ryan & Wilson (2008)	medicine
Sonnichsen (2019)	
Hill et al (2020)	No mention
Verzantvoort et al (2018)	health sciences
Morita et al (2017)	medicine
Millenson et al (2018)	medicine
Aboueid et al (2019)	health sciences
Chambers et al (2019)	health sciences
Kujala et al (2022)	computer science
Hagemen et al (2015)	medicine
Kao & Liebovitz (2017)	medicine
Kuhn et al (2018)	
Iacobucci (2020)	No mention
Semigran et al (2015)	medicine
Wyatt (2015)	health sciences
Lanseng & Andreassen (2007)	marketing
Herzog (2019)	engineering
Wattanapisit et al (2020)	medicine
Razzaki et al (2018)	No mention
Meyer et al (2020)	medicine
Miller et al (2020)	No mention
Thielscher and Antes (2019)	
Copeland et al (2018)	computer science
Nijland et al (2010)	psychology
Shen et al (2019)	medicine
Jimison et al (2007)	informatics
Marco-Luiz et al (2017)	medicine
Luger et al (2014)	medicine
Albrecht et al (2016)	
Rowland et al (2020)	medicine
Palanica et al (2019)	No mention
Chambers et al (2019)	health sciences
Blease et al (2019)	medicine
Denecke et al (2019)	No mention
Summerton & Cansdale (2019)	medicine
Semigran et al (2015)	medicine
Jutel & Lupton (2015)	nursing
Lupton et al (2015)	social sciences
Gottliebsen & Petersson (2020)	medicine
Kujala et al (2022)	computer science
Miles et al (2021)	public health
Wattanapisit et al (2020)	medicine
Kuhn et al (2018)	
Muschenich et al (2018)	
Merz et al (2018)	
Powley et al (2016)	medicine
Bisson et al (2014)	medicine
Bisson et al (2016)	medicine

Hagemen et al (2015)	medicine
Knitza et al (2021)	medicine
Cross et al (2021)	No mention
Verzantvoort et al (2018)	health sciences
Fan et al (2021)	computer science
Prince et al (2013)	medicine
You & Gui (2021)	health informatics
Aboueid et al (2021)	public health
Aboueid et al (2021)	public health
Marco-Luiz et al (2017)	medicine
Ponnada (2020)	informatics
Schrager et al (2020)	medicine
Reilly & Austin (2021)	health sciences
Nieroda et al (2018)	marketing
Daher et al (2020)	No mention
Tsai et al., (2022)	information science
Li et al (2020)	computer science
You et al (2021)	informatics
Hwang et al (2018)	No mention
Baldauf et al (2020)	health sciences
Miller et al (2020)	No mention
Meyer et al (2020)	medicine
Winn et al (2019)	medicine
DeForte et al (2020)	medicine
Nijland et al (2010)	psychology
Luger et al (2014)	medicine
Lanseng & Andreassen (2007)	marketing
Hua & Hou (2020)	business
Semigran et al (2015)	medicine
Poote et al (2014)	psychology
Semigran et al (2016)	medicine
Verzantvoort et al (2018)	health sciences
Berry et al (2019)	medicine
Gilbert et al (2020)	No mention
Hill et al (2020)	No mention
Yu et al (2021)	medicine
Ceney et al (2021)	No mention
Chan et al (2021)	medicine
Delshad et al (2021)	medicine
Gilbert et al (2021)	No mention
Trivedi et al (2020)	medicine
Dickson et al (2022)	medicine
Aboueid et al (2019)	health sciences

***The review by Char et al., 2019 focuses on machine l[illegible]**
gerenal, without a specific focus on self-diagnosis. How[illegible]
this is why it could not be exluded from the review (bas[illegible]
to have reviewed 83 articles, whose citations cannot[illegible]
reference list contains only 58 articles... Given that it is [illegible]
were exclusively about self-diagnosis, the papers by Ch[illegible]
here.

PAPER INFORMATION

journal	title
journal of medical internet research	Triage Accuracy of Symptom Checker
journal of medical internet research	Health Care Professionals' Experience
conference paper	Exploring and Promoting Diagnostic Tı
european journal of emergency medicine	Agreement and validity of electronic p
plos one	Performance of a new symptom checl
Health Services and Delivery Research	Impact of NHS 111 Online on the NHS
journal of medical internet research	Triage Accuracy of Symptom Checker
hong kong journal of emergency medicine	Triage accuracy of online symptom ch
amia annual symposium proceedings archive	Self-diagnosis through AI-enabled cha
jmir public health and surveillance	Young Adults' Perspectives on the Use
proceedings of the web conference	Search engines vs. symptom checkers
journal of medical internet research	Use Characteristics and Triage Acuity (
journal of medical internet research	Benchmarking Triage Capability of Syr
medical journal of australia	The quality of diagnosis and triage adv
bmj health & care information	Limited evidence of benefits of patien
bcm medical informatics and decision making	Thailand medical mobile application fo
british journal of general practice	Digital interventions for parents of acu
journal of medical internet research	Patient Perspectives on the Usefulnes
bmj open	Digital and online symptom checkers a
jmir medical informatics	The Use of Artificially Intelligent Self-D
de gruyter	Beyond Dr. Google: the evidence on c
plos one	Self-triage for acute primary care via a
No mention	Detection of patterns and trends in pa
bmj open	The impact of demand management s
epidemiology & infection	Internet-based remote health self-che
bmj	Evaluation of symptom checkers for se
social science & medicine	'It's like having a physician in your poc
journal of telemedicine and telecare	Patient use and compliance with med
babylon health	NHS111 powered by babylon: outcom
gastroenterology	Symptom Checkers vs. Doctors, the Ul
american journal of gastroenterology	Symptom Checkers versus Doctors: A
gastroenterology	Symptom Checkers vs. Doctors, the Ul
annals of emergency medicine	Web-Based Self-Triage of Influenza-Lil
bmj open	Primary care randomised controlled tı
journal of medical internet research	Older Adult Experience of Online Diag
journal of biomedical informatics	Combining multivariate statistics and
pre-print / babylon health	Sorting out symptoms: design and eva
informatics in primary care	through telephony and web
journal of telemedicine and telecare	Patient use and compliance with med
journal of telemedicine and telecare	A study of automated self-assessment
jama pediatrics	Feasibility of Web-Based Self-Triage b
jama international medicine	Comparison of Physician and Compute
bmj	Evaluation of symptom checkers for se
journal of american college of health	Web-Based Triage in a College Health
journal of medical internet research	Evaluation of a Web-based Interventio
bmj open	Feasibility, acceptability and effectiver

enviromental research and public health	Evaluation of a Digital Consultation an
web GP	*WebGP: the Virtual general practice*
NHS England	*NHS111 online evaluation*
journal of medical informatics	Increasing the use of e-consultation in
International Journal of Service Industry Manage	Electronic Healthcare: A Study of Peop
american journal of sports medicine	Accuracy of a Computer-Based Diagno
Orthopaedic Journal of Sports Medicine	How Accurate Are Patients at Diagnos
10th International Conference on e-Health	m-Health application Interface design
clinical otolaryngology	How good is Internet self-diagnosis of
europe pmc	Internet self-diagnosis in hand surgery
International Journal of Service Industry Manage	Electronic healthcare: a study of peop
journal of medical internet research	Older Adult Experience of Online Diag
bmc musculoskeletal disorders	Are online symptoms checkers useful
journal of medical internet research	A Novel Insight Into the Challenges of
europe pmc	Reducing diagnostic errors in primary
europe pmc	Ethical perspectives on recommendin
No mention	Regulations for eHealth & mHealth Ap
online journal of public health informatics	Mobile medical and health apps: state
american journal of law & medicine	Digital diagnosis: privacy and the regu
de gruyter	Digitizing diagnosis: a review of mobil
clinical informatics in physiatry	Consumer Mobile Health Apps: Curren
social science & medicine	'It's like having a physician in your poc
health policy and management	The Potential Possibility of Symptom C
expert opinion on drug safety	Internet healthcare: do self-diagnosis
bmj	Evaluation of symptom checkers for se
europe pmc	Internet self-diagnosis in hand surgery
epidemiology & infection	Online symptom checker diagnostic an
medical journal of australia	The quality of diagnosis and triage adv
jama ophthalmology	Accuracy of a Popular Online Symptom
hong kong journal of emergency medicine	Triage accuracy of online symptom ch
IJIMAI	Evaluation of a diagnostic decision sup
bmc musculoskeletal disorders	Are online symptoms checkers useful
journal of prosthodontic research	Accuracy of online symptom checkers
bmj open	How accurate are digital symptom ass
lancet	Safety of patient-facing digital sympto
The Guide to Health Informatics	Paper Review: the Babylon Chatbot
babylon health	intelligence and human
Stud Health Technol Inform	Artificial intelligence powered sympto
de gruyter	Beyond Dr. Google: the evidence on c
enviromental research and public health	Evaluation of a Digital Consultation an
bmj open	Feasibility, acceptability and effective
bmj open	Use of a primary care online consultat
british journal of general practice	Experiences with online consultation s
bmj open	on
bmj	Evaluation of symptom checkers for se
jama international medicine	Comparison of Physician and Compute
diagnosis	Digitizing diagnosis: a review of mobil
patient education and counseling	How to develop web-based decision s

journal of telemedicine and telecare	Patient use and compliance with med
journal of medical informatics	Increasing the use of e-consultation in
Medicine Meets Virtual Reality	Decision Support System for Medical T
bmj	Evaluation of symptom checkers for s
bmj open	How accurate are digital symptom ass
bmj health & care informatics	Limited evidence of benefits of patien
bmj open	Digital and online symptom checkers a
pre-print / babylon health	Sorting out symptoms: design and eva
book chapter	Quro: Facilitating user symptom check
babylon health	[illegible] intelligence and human
jmir medical informatics	Human- Versus Machine Learning–Bas
epidemiology & infection	Online symptom checker diagnostic an
american journal of sports medicine	Accuracy of a Computer-Based Diagno
Orthopaedic Journal of Sports Medicine	How Accurate Are Patients at Diagnos
journal of medical internet research	A Novel Insight Into the Challenges of
journal of medical internet research	A Brief Web-Based Screening Question
clinical otolaryngology	How good is Internet self-diagnosis of
journal of medical internet research	A Web-Based Screening Instrument fo
journal of american academy of dermalogy	Skin scan: A demonstration of the nee
europe pmc	Internet self-diagnosis in hand surgery
cardiovascular and metabolic risk	Diabetes Risk Calculator: A simple too
Health Recommender Systems	Personalized symptom checker using r
IEEE access	[illegible] Technique Exploiting Automatic
the medical journal of australia	Online chlamydia testing: an innovativ
journal of general internal medicine	Making Sense of Cancer Risk Calculato
journal of medical systems	The 'lottery' of cardiovascular risk esti
BMC Musculoskeletal Disorders	Development and feasibility of a perso
arthritis care & research	Randomized Controlled Trial of an Edu
journal of european academy of dermatology	Accuracy of a smartphone application
pre-print / babylon health	Sorting out symptoms: design and eva
journal of telemedicine and telecare	Patient use and compliance with med
australian journal of dermatology	Efficacy of smartphone applications in
journal of telemedicine and telecare	A study of automated self-assessment
bmc musculoskeletal disorders	Are online symptoms checkers useful
jama pediatrics	Feasibility of Web-Based Self-Triage b
bmj	Evaluation of symptom checkers for s
pre-print	Interactive Symptom Elicitation for Di
jama ophthalmology	Accuracy of a Popular Online Symptom
journal of american college of health	Web-Based Triage in a College Health
social science & medicine	The double-edged sword of digital sel
Medical Journal of Australia	Will online symptom checkers improv
lancet	Safety of patient-facing digital sympto
de gruyter	Digitizing diagnosis: a review of mobil
social science & medicine	'It's like having a physician in your poc
bmc musculoskeletal disorders	Are online symptoms checkers useful

expert opinion on drug safety	Internet healthcare: do self-diagnosis
medical journal of australia	The quality of diagnosis and triage adv
plos one	Self-triage for acute primary care via a
health policy and management	The Potential Possibility of Symptom (
de gruyter	Beyond Dr. Google: the evidence on c
jmir medical informatics	The Use of Artificially Intelligent Self-D
bmj open	Digital and online symptom checkers a
journal of medical internet research	Health Care Professionals' Experience
europe pmc	Internet self-diagnosis in hand surgery
clinical informatics in physiatry	Consumer Mobile Health Apps: Curren
bmj	Row over Babylon's chatbot shows lac
bmj	Evaluation of symptom checkers for se
bmj	Fifty million people use computerised
International Journal of Service Industry Manage	Electronic Healthcare: A Study of Peop
pre print	Technological opacity of machine lear
bmc medical informatics	Can mobile health apps replace GPs? A
babylon health	intelligence and human
journal of medical internet research	Patient Perspectives on the Usefulnes
jmir human factors	Patients' Utilization and Perception of
10th International Conference on e-Health	m-Health application Interface design
journal of telemedicine and telecare	Patient use and compliance with med
jama ophthalmology	Accuracy of a Popular Online Symptor
book chapter	Decision support for patients
journal of biomedical informatics	Combining multivariate statistics and
journal of medical internet research	Older Adult Experience of Online Diag
digital medicine	What is the clinical value of mHealth for patier
journal of medical internet research	Physicians' Perceptions of Chatbots in
bmj open	Digital and online symptom checkers a
journal of medical internet research	Artificial Intelligence and the Future o
book chapter	Intelligent conversational agents in he
British Journal of General Practice	in general practice
bmj	Evaluation of symptom checkers for se
de gruyter	Digitizing diagnosis: a review of mobile
social science & medicine	'It's like having a physician in your poc
bmj health & care informatics	Limited evidence of benefits of patien
journal of medical internet research	Health Care Professionals' Experience
digital health	Health chatbots acceptability modera
bmc medical informatics	Can mobile health apps replace GPs? A
bmc musculoskeletal disorders	Are online symptoms checkers useful
american journal of sports medicine	Accuracy of a Computer-Based Diagno
Orthopaedic Journal of Sports Medicine	How Accurate Are Patients at Diagnos

europe pmc	Internet self-diagnosis in hand surgery
arthritis research & therapy	Accuracy, patient-perceived usability,
proceedings of the web conference	Search engines vs. symptom checkers
plos one	Self-triage for acute primary care via a
journal of internet medical research	Utilization of Self-Diagnosis Health Ch
jama pediatrics	Feasibility of Web-Based Self-Triage b
amia annual symposium proceedings archive	Self-diagnosis through AI-enabled cha
jmir public health and surveillance	Young Adults' Perspectives on the Use
bmj	Use of symptom checkers for COVID-1
journal of biomedical informatics	Combining multivariate statistics and
pre print	Reimagining the COVID-19 Digital Exp
emergency medicine	Development and Usability Testing of
journal of medical internet research	Attitudes and Engagement of Pregnan
jmir cancer	Online Decision Support Tool for Pers
conference paper	Empathic Chatbot Response for Medic
conference paper	Exploring and Promoting Diagnostic Tr
conference paper	Research on the Influencing Factors
conference paper	of AI-enabled
conference paper	Leveraging challenges of an<br>algorithm-based symptom checker
journal of medical internet research	Trust Me, I'm a Chatbot: How<br>Artificial Intelligence in Health Care
jmir human factors	Patients' Utilization and Perception of
journal of medical internet research	Patient Perspectives on the Usefulnes
jame network open	Symptom Checkers With Patients'
jmir mhealth and uhealth	The Association Between App<br>Administered Depression
journal of telemedicine and telecare	Patient use and compliance with med
journal of medical internet research	Older Adult Experience of Online Diag
International Journal of Service Industry Manage	Electronic Healthcare: A Study of Peop
Journal of Health, Medicine and Nursing	Factors That Influence the Intention<br>to Use Self-Diagnosis Apps in
bmj	Evaluation of symptom checkers for s
journal of telemedicine and telecare	A study of automated self-assessment
jama international medicine	Comparison of Physician and Comput
plos one	Self-triage for acute primary care via a
epidemiology & infection	Online symptom checker diagnostic a
bmj open	How accurate are digital symptom ass
medical journal of australia	The quality of diagnosis and triage adv
hong kong journal of emergency medicine	Triage accuracy of online symptom ch
plos one	Accuracy of online symptom checkers
plos one	Performance of a new symptom check
europe pmc	Artificial Intelligence-Based Applicatio
australian journal of primary care	[illegible]<br>urgency advice provided
europe pmc	A Comparison Between Computer-Ass
european journal of emergency medicine	Agreement and validity of electronic p
jmir medical informatics	The Use of Artificially Intelligent Self-D

earning applications in healthcare, in
ever, they do mention self-diagnosis so
ed on our inclusion criteria). They claim
: be accessed. However, the paper's
not possible to figute out which papers
ar et al., 2019 have not been included

type	review
empirical study	Pairon et al., 2023
empirical study	Pairon et al., 2023
empirical study	Pairon et al., 2023
empirical study	Pairon et al., 2023
empirical study	Pairon et al., 2023
empirical study	Pairon et al., 2023
empirical study	Pairon et al., 2023
empirical study	Pairon et al., 2023
review	Pairon et al., 2023
qualitative study	Pairon et al., 2023
empirical study	Pairon et al., 2023
empirical study	Pairon et al., 2023
empirical study	Pairon et al., 2023
empirical study	Pairon et al., 2023
review	Pairon et al., 2023
empirical study	Pairon et al., 2023
review	Pairon et al., 2023
empirical study	Pairon et al., 2023
review	Pairon et al., 2023
review	Pairon et al., 2023
review	Pairon et al., 2023
empirical study	Pairon et al., 2023
empirical study	Pairon et al., 2023
empirical study	Pairon et al., 2023
empirical study	Pairon et al., 2023
empirical study	Pairon et al., 2023
qualitative study	Pairon et al., 2023
empirical study	Pairon et al., 2023
empirical study	Chambers et al., 2019
empirical study	Chambers et al., 2019
empirical study	Chambers et al., 2019
empirical study	Chambers et al., 2019
empirical study	Chambers et al., 2019
empirical study	Chambers et al., 2019
empirical study	Chambers et al., 2019
empirical study	Chambers et al., 2019
empirical study	Chambers et al., 2019
empirical study	Chambers et al., 2019
empirical study	Chambers et al., 2019
empirical study	Chambers et al., 2019
empirical study	Chambers et al., 2019
empirical study	Chambers et al., 2019
empirical study	Chambers et al., 2019
empirical study	Chambers et al., 2019
empirical study	Chambers et al., 2019
empirical study	Chambers et al., 2019

empirical study	Chambers et al., 2019
empirical study	Chambers et al., 2019
empirical study	Chambers et al., 2019
empirical study	Chambers et al., 2019
empirical study	Chambers et al., 2019
empirical study	Aboueid et al., 2019
empirical study	Aboueid et al., 2019
empirical study	Aboueid et al., 2019
review	Aboueid et al., 2019
empirical study	Aboueid et al., 2019
empirical study	Aboueid et al., 2019
empirical study	Aboueid et al., 2019
empirical study	Aboueid et al., 2019
empirical study	Aboueid et al., 2019
review	Aboueid et al., 2019
review	Aboueid et al., 2019
review	Aboueid et al., 2019
review	Aboueid et al., 2019
review	Aboueid et al., 2019
review	Aboueid et al., 2019
review	Aboueid et al., 2019
qualitative study	Aboueid et al., 2019
review	Aboueid et al., 2019
review	Aboueid et al., 2019
empirical study	Wallace et al., 2022
empirical study	Wallace et al., 2022
empirical study	Wallace et al., 2022
empirical study	Wallace et al., 2022
empirical study	Wallace et al., 2022
empirical study	Wallace et al., 2022
empirical study	Wallace et al., 2022
empirical study	Wallace et al., 2022
empirical study	Wallace et al., 2022
empirical study	Wallace et al., 2022
empirical study	Gottliebsen & Petersson, 2020
review	Gottliebsen & Petersson, 2020
empirical study	Gottliebsen & Petersson, 2020
empirical study	Gottliebsen & Petersson, 2020
review	Gottliebsen & Petersson, 2020
empirical study	Gottliebsen & Petersson, 2020
empirical study	Gottliebsen & Petersson, 2020
empirical study	Gottliebsen & Petersson, 2020
empirical study	Gottliebsen & Petersson, 2020
empirical study	Gottliebsen & Petersson, 2020
empirical study	Gottliebsen & Petersson, 2020
empirical study	Gottliebsen & Petersson, 2020
review	Gottliebsen & Petersson, 2020
review	Gottliebsen & Petersson, 2020

empirical study	Gottliebsen & Petersson, 2020
empirical study	Gottliebsen & Petersson, 2020
empirical study	Gottliebsen & Petersson, 2020
empirical study	Illicki, 2022
empirical study	Illicki, 2022
review	Illicki, 2022
review	Illicki, 2022
empirical study	Illicki, 2022
empirical study	Illicki, 2022
empirical study	Illicki, 2022
empirical study	Illicki, 2022
empirical study	Jovicic, 2020
empirical study	Jovicic, 2020
empirical study	Jovicic, 2020
empirical study	Jovicic, 2020
empirical study	Jovicic, 2020
review	Jovicic, 2020
empirical study	Jovicic, 2020
review	Jovicic, 2020
empirical study	Jovicic, 2020
empirical study	Jovicic, 2020
empirical study	Jovicic, 2020
empirical study	Jovicic, 2020
empirical study	Jovicic, 2020
review	Jovicic, 2020
review	Jovicic, 2020
empirical study	Jovicic, 2020
empirical study	Jovicic, 2020
empirical study	Jovicic, 2020
empirical study	Jovicic, 2020
empirical study	Jovicic, 2020
empirical study	Jovicic, 2020
empirical study	Jovicic, 2020
empirical study	Jovicic, 2020
empirical study	Jovicic, 2020
empirical study	Jovicic, 2020
empirical study	Jovicic, 2020
empirical study	Jovicic, 2020
empirical study	Jovicic, 2020
qualitative study	Muller et al, 2022
review	Muller et al, 2022
empirical study	Muller et al, 2022
review	Muller et al, 2022
qualitative study	Muller et al, 2022
	Muller et al, 2022
	Muller et al, 2022
empirical study	Muller et al, 2022

review	Muller et al, 2022
	Muller et al, 2022
empirical study	Muller et al, 2022
empirical study	Muller et al, 2022
review	Muller et al, 2022
review	Muller et al, 2022
review	Muller et al, 2022
review	Muller et al, 2022
empirical study	Muller et al, 2022
empirical study	Muller et al, 2022
review	Muller et al, 2022
	Muller et al, 2022
review	Muller et al, 2022
empirical study	Muller et al, 2022
review	Muller et al, 2022
empirical study	Muller et al, 2022
review	Muller et al, 2022
review	Muller et al, 2022
empirical study	Muller et al, 2022
empirical study	Muller et al, 2022
empirical study	Muller et al, 2022
	Muller et al, 2022
empirical study	Muller et al, 2022
empirical study	Muller et al, 2022
empirical study	Muller et al, 2022
review	Muller et al, 2022
empirical study	Muller et al, 2022
empirical study	Muller et al, 2022
	Muller et al, 2022
review	Muller et al, 2022
empirical study	Radionova et al., 2023
review	Radionova et al., 2023
qualitative study	Radionova et al., 2023
althcare: hype or hope?	Radionova et al., 2023
review	Radionova et al., 2023
empirical study	Radionova et al., 2023
review	Radionova et al., 2023
qualitative study	Radionova et al., 2023
review	Radionova et al., 2023
empirical study	Radionova et al., 2023
empirical study	Radionova et al., 2023
review	Radionova et al., 2023
	Radionova et al., 2023
	Radionova et al., 2023
	Radionova et al., 2023
empirical study	You et al., 2022
empirical study	You et al., 2022
empirical study	You et al., 2022

empirical study	You et al., 2022
empirical study	You et al., 2022
empirical study	You et al., 2022
empirical study	You et al., 2022
empirical study	You et al., 2022
empirical study	You et al., 2022
review	You et al., 2022
qualitative study	You et al., 2022
qualitative study	You et al., 2022
empirical study	You et al., 2022
empirical study	You et al., 2022
empirical study	You et al., 2022
empirical study	You et al., 2022
empirical study	You et al., 2022
empirical study	You et al., 2022
empirical study	You et al., 2022
empirical study	You et al., 2022
empirical study	You et al., 2022
empirical study	You et al., 2022
review	You et al., 2022
empirical study	You et al., 2022
empirical study	You et al., 2022
empirical study	You et al., 2022
empirical study	You et al., 2022
empirical study	You et al., 2022
empirical study	You et al., 2022
empirical study	You et al., 2022
empirical study	You et al., 2022
empirical study	Riboli-Sasco et al., 2023
empirical study	Riboli-Sasco et al., 2023
empirical study	Riboli-Sasco et al., 2023
empirical study	Riboli-Sasco et al., 2023
empirical study	Riboli-Sasco et al., 2023
empirical study	Riboli-Sasco et al., 2023
empirical study	Riboli-Sasco et al., 2023
empirical study	Riboli-Sasco et al., 2023
empirical study	Riboli-Sasco et al., 2023
empirical study	Riboli-Sasco et al., 2023
empirical study	Riboli-Sasco et al., 2023
empirical study	Riboli-Sasco et al., 2023
empirical study	Riboli-Sasco et al., 2023
empirical study	Riboli-Sasco et al., 2023
review	Char et al., 2019

	**terms used: tools**
	symptom checker apps
	web-based symptom checkers
	online symptom checkers
NO ACCESS FULL TEXT	electronic patient self-triage (eTriage)
	symptom checkers
	NHS 111 Online (symptom checker)
	symptom checker apps
	online symptom checkers
	AI-enabled chatbot-based symptom checker
	symptom checkers
	online symptom checkers
	digital symptom checkers /symptom checker
	symptom checkers
	online symptom checker apps
	intelligent online triage tools
	medical triage mobile application
	digital interventions
	Artificial Intelligence–Assisted Symptom Che
	digital or online symptom checker or health
	Artificially Intelligent Self-Diagnosing Digital I
	consumer-facing digital tools for diagnosis
	smartphone application
NO ACCESS FULL TEXT	electronic applications and Internet resource
	No mention
	national internet-based 'symptom checker' s
	online symptom checkers
	self-diagnosis smartphone apps
	web-based triage system
	NHS 111 Online (symptom checker)
	symptom checkers
NO ACCESS FULL TEXT	symptom checkers
	symptom checkers
	Web-Based Self-Triage
	internet-delivered intervention providing adv
	Online Diagnosis
	symptom checkers
	automated triage system/ automated sympt
	self-management webpages
	web-based triage system
	self-assessment triage system
	web-based decision support tool
	sumptom checkers
	online symptom checkers
NO ACCESS FULL TEXT	Web-based triage system
	Internet Doctor webpage (online self-manag
	webGP

	eConsult, a web-based triage and consultatic
	symptom checkers
NO ACCESS FULL TEXT	NHS 111 Online (symptom checker)
	e-consultation in primary care
	self diagnosis technology
	web-based symptom-checking programs
	web-based symptom checker programs
	mHealth symptom checkers
	Boots WebMD symptom checker
	Internet self-diagnosis / symptom checkers
NO ACCESS FULL TEXT	internet-based medical self-diagnosis applica
	Online Diagnosis
	symptom checkers
	Web-based symptom checkers
	Computerized diagnostic decision support sy
	online self-diagnosis/ symptom checkers
	eHealth & mHealth apps
	mobile clinical and health-related apps
NO ACCESS FULL TEXT	
	mobile apps
	consumer-facing applications (apps)/ mobile
	self-diagnosis smartphone apps
	symptom checkers
NO ACCESS FULL TEXT	self-diagnosis websites
	symptom checkers
	Internet self-diagnosis / symptom checkers
	symptom checkers
	online symptom checker apps
NO ACCESS FULL TEXT	online symptom checkers
	online symptom checkers
	Diagnostic decision support systems
	symptom checkers
	online symptom checkers
	digital symptom assessment apps
	Computerised diagnostic decision support (C
NO ACCESS FULL TEXT	
	online symptom checkers
	automated chatbot for triage and conditions assessment, based on user
	consumer-facing digital tools for diagnosis
	eConsult, a web-based triage and consultatic
	webGP
	a primary care online consultation system
	an online consultation system (Tele-Doc)
	a nationwide evidence-based health website
	online symptom checkers
	sumptom checkers
	mobile apps
	web-based decision support interventions fo

	web-based triage system
	e-consultation in primary care
NO ACCESS FULL TEXT	
	online symptom checkers
	digital symptom assessment apps (i.e., Ada, I
	atient operated intelligent primary care triag
	digital or online symptom checker or health
	automated triage system/ automated sympt
	automated chatbot for triage and conditions
	online symptom checkers
	automated ML-based triage method
	symptom checkers
	web-based symptom-checking programs
	web-based symptom checker programs
	Web-based symptom checkers
	Web Screening Questionnaire (WSQ)
	Boots WebMD symptom checker
	Web-based, self-report screener for major d
	medical apps
	Internet self-diagnosis / symptom checkers
	Diabetes Risk Calculator
	symptom checker
	online health applications
	No mention
	risk calculators
	risk calculators on the Internet
	Risk calculators
	risk calculator
	risk calculator
	automated triage system/ automated sympt
	web-based triage system
	risk calculators
	self-assessment triage system
	symptom checkers
	web-based decision support tool
	online symptom checkers
	interactive symptom elicitation
NO ACCESS FULL TEXT	online symptom checkers
NO ACCESS FULL TEXT	Web-based triage system
	Digital self-care
	online symptom checkers
	Computerised diagnostic decision support (C
	mobile apps
	self-diagnosis smartphone apps
FOREIGN LANGUAGE	
NO ACCESS FULL TEXT	
	symptom checkers

NO ACCESS FULL TEXT	self-diagnosis websites
FOREIGN LANGUAGE	
	online symptom checker apps
	smartphone application
	symptom checkers
	consumer-facing digital tools for diagnosis
	Artificially Intelligent Self-Diagnosing Digital I
	digital or online symptom checker or health a
	web-based symptom checkers
	Internet self-diagnosis / symptom checkers
	consumer-facing applications (apps)/ mobile
FOREIGN LANGUAGE	
	Babylon's chatbot
	online symptom checkers
	computerised self triage
	self diagnosis technology
	No mention
	Mobile health applications (mHealth apps)
	online symptom checkers
	Artificial Intelligence–Assisted Symptom Che
	Artificial Intelligence–Based Symptom Assess
FOREIGN LANGUAGE	
	mHealth symptom checkers
	web-based triage system
NO ACCESS FULL TEXT	online symptom checkers
	computer-based decision support systems
	symptom checkers
	Online Diagnosis
FOREIGN LANGUAGE	
	mHealth apps
	health care chatbots
	digital or online symptom checker or health a
	No mention
	intelligent conversational agents in
	AI-based symptom checkers
	online symptom checkers
	mobile apps
	self-diagnosis smartphone apps
	atient operated intelligent primary care triag
	web-based symptom checkers
	Chatbots and virtual voice assistants
	Mobile health applications (mHealth apps)
FOREIGN LANGUAGE	
FOREIGN LANGUAGE	
NO ACCESS FULL TEXT	
	symptom checkers
	web-based symptom-checking programs
	web-based symptom checker programs

Internet self-diagnosis / symptom checkers
symptom checkers
online symptom checkers
smartphone application
Self-Diagnosis Health Chatbots
web-based decision support tool
AI-enabled chatbot-based symptom checker
symptom checkers
symptom checkers
symptom checkers
COVID-19 symptom self checker
Web-based COVID-19 Self-triage Platform
Web-Based Emotional Health Tool
Risk Assessment Tools (RATs) and QCancer
medical assistant chatbots
online symptom checkers
intelligent self diagnosis system
Artifcial Intelligence (AI)-based symptom che
lgorithm-based symptom checker
user-facing artificial intelligence systems in h
Artificial Intelligence–Based Symptom Assess
Artificial Intelligence–Assisted Symptom Che
Online triage tools
App-Administered Depression Assessments
web-based triage system
Online Diagnosis
self diagnosis technology
self-diagnostic apps
online symptom checkers
self-assessment triage system
sumptom checkers
smartphone application
symptom checkers
digital symptom assessment apps
online symptom checker apps
online symptom checkers
online symptom checkers
symptom checkers
AI-based application, MayaMD
Ada symptom assessment app
Computer-Assisted Self-Triage
NO ACCESS FULL TEXT electronic patient self-triage (eTriage)
Artificially Intelligent Self-Diagnosing Digital I

tool categorisation	definition
symptom checker	digital tools assisting laypersons in
symptom checker	tools that provide help to patients se
symptom checker	intelligent systems that
triage	
symptom checker	symptom checkers aim to help patie
symptom checker	A web-based version of
symptom checker	digital tools assisting laypersons in
symptom checker	programs that provide t
symptom checker	A chatbot is defined as a
symptom checker	Using a question-and-answer chat f
symptom checker	Symptom checkers are c
symptom checker	Digital symptom checke
symptom checker	tools developed to prov
symptom checker	Symptom checkers (SCs)
triage	the main component of
triage	enabling patients to tria
digital intervention	providing evidence-base
symptom checker	Patients research their s
symptom checker	symptom checkers and
self-diagnosis	On the basis of the med
self-diagnosis	“App” defined as intera
triage	advise patients whether
self-diagnosis	
NA	No mention
symptom checker	a series of clinical assess
symptom checker	tools that use computer
self-diagnosis	No mention
triage	The symptom-driven tria
symptom checker	No mention
symptom checker	No mention
symptom checker	
symptom checker	No mention
triage	No mention
self-management	users could select tailore
self-diagnosis	Searching for informatio
symptom checker	software tools that allo
triage	provide a differential
self-management	No mention
triage	The symptom-driven tria
triage	Patients had a simple us
triage	designed to translate cli
symptom checker	Symptom checkers are v
symptom checker	tools that use computer
triage	
self-management	web-based support for self-care in t
e-consultation	suite of five services

e-consultation	
symptom checker	help patients confirm their GP is the right
symptom checker	
e-consultation	Direct e-consultation:
self-diagnosis	technological interfaces
symptom checker	Internet-based program
symptom checker	Briefly, after entry of sy
symptom checker	No mention
symptom checker	No mention
self-diagnosis	The WebMD Symptom C
self-diagnosis	
self-diagnosis	Searching for informatio
symptom checker	Symptom checker type v
symptom checker	websites that allow pati
clinical decision support	Computerized diagnosti
symptom checker	No mention
mobile health	No mention
mobile health	No mention
self-diagnosis	
self-diagnosis	apps that focused speci
mobile health	Mobile health (mHealth)
self-diagnosis	No mention
symptom checker	Symptom checkers are a
self-diagnosis	
symptom checker	tools that use computer
self-diagnosis	The WebMD Symptom C
symptom checker	checkers are
symptom checker	Symptom checkers (SCs)
symptom checker	
symptom checker	programs that provide t
clinical decision support	Along with this approach, there has
symptom checker	Symptom checker type v
symptom checker	Symptom checkers are s
symptom checker	assessment
clinical decision support	Originally designed for d
symptom checker	checkers are a
symptom checker	
self-diagnosis	“App” defined as intera
e-consultation	
e-consultation	suite of five services
e-consultation	eConsult (hereafter refe
e-consultation	No mention
mobile health	The primary goal of the v
symptom checker	tools that use computer
symptom checker	Symptom checkers are v
self-diagnosis	apps that focused speci
decision support (patient-facing)	No mention

triage	The symptom-driven tri
e-consultation	Direct e-consultation:
symptom checker	tools that use computer
symptom checker	use a structured intervie
triage	Digitalisation include the
symptom checker	symptom checkers and
symptom checker	provide a differential
triage	engages patients in
symptom checker	checkers are a
triage	The automated patient interviewing software
symptom checker	checkers are
symptom checker	Internet-based program
symptom checker	Briefly, after entry of sy
symptom checker	websites that allow pati
screening	we developed a brief on
symptom checker	No mention
screening	This test, the Web-Base
mobile health	No mention
symptom checker	The WebMD Symptom C
risk calculator	
symptom checker	which aims to
self-diagnosis	an intelligent health dia
screening	No mention
risk calculator	These tools ask the site
risk calculator	No mention
risk calculator	Risk estimates for calcul
risk calculator	provide patients with ac
risk calculator	To determine the risk as
triage	provide a differential
triage	The symptom-driven tri
risk calculator	they provided an overall
triage	Patients had a simple us
symptom checker	Symptom checker type
triage	designed to translate cli
symptom checker	tools that use computer
symptom checker	elicitation builds a
symptom checker	
triage	
mobile health	practices of digital self-c
symptom checker	an online symptom chec
symptom checker	Originally designed for d
self-diagnosis	apps that focused speci
self-diagnosis	No mention
symptom checker	Symptom checker type

self-diagnosis	
symptom checker	Symptom checkers (SCs)
triage	advise patients whether
symptom checker	Symptom checkers are a
self-diagnosis	“App” defined as intera
self-diagnosis	On the basis of the med
symptom checker	symptom checkers and
symptom checker	tools that provide help to patients se
symptom checker	The WebMD Symptom C
mobile health	Mobile health (mHealth)
triage	symptom checker
symptom checker	tools that use computer
triage	No mention
self-diagnosis	technological interfaces
NA	No mention
mobile health	Mobile apps are a vital c
symptom checker	checkers are a
symptom checker	Patients research their s
symptom checker	digital symptom checker
symptom checker	No mention
triage	The symptom-driven tria
symptom checker	
decision support (patient-facing)	systems have been
symptom checker	software tools that allo
self-diagnosis	Searching for informatio
symptom checker	Diagnostic symptom che
triage	Chatbots, also known as
symptom checker	symptom checkers and
NA	No mention
NA	New developments in
symptom checker	improving the accuracy
symptom checker	tools that use computer
self-diagnosis	apps that focused speci
self-diagnosis	No mention
triage	Digitalisation include the
symptom checker	tools that provide help to patients se
triage	Conversational agents, c
mobile health	Mobile apps are a vital c
symptom checker	Symptom checker type v
symptom checker	Internet-based program
symptom checker	Briefly, after entry of sy

self-diagnosis	The WebMD Symptom C
symptom checker	Symptom checkers (SCs)
symptom checker	Symptom checkers are c
triage	advise patients whether
self-diagnosis	That is, AI-driven intellig
decision support (patient-facing)	designed to translate cli
symptom checker	A chatbot is defined as a
symptom checker	Using a question-and-answer chat f
symptom checker	in
symptom checker	software tools that allo
symptom checker	and guide the general
triage	web-based, self-triage to
screening	tool that allows women
risk assessment	RATs consider only symp
mobile health	provid ing a diagnosis
symptom checker	intelligent systems that
self-diagnosis	no mention
symptom checker	information from users
symptom checker	symptom checker is a
chatbot	applications and
symptom checker	digital symptom checker
symptom checker	Patients research their s
triage	available virtual triage
screening	No mention
triage	The symptom-driven tria
self-diagnosis	Searching for informatio
self-diagnosis	technological interfaces
self-diagnosis	No mention
self-diagnosis	tools that use computer
triage	Patients had a simple us
symptom checker	Symptom checkers are v
triage	advise patients whether
symptom checker	checkers are
symptom checker	assessment
symptom checker	Symptom checkers (SCs)
symptom checker	programs that provide tr
symptom checker	The current available sym
symptom checker	symptom checkers aim to help patie
self-diagnosis	MayaMD is an AI-based
symptom checker	applications
triage	No mention
triage	
self-diagnosis	On the basis of the med

VARIABLES

N of unique terms: tools	terms used: AI	definition
1	No mention	No mention
1	algorythms	Algorithm-assisted symptom check
1	explainable AI	explainable AI, which foc
1		
1	Computerized algorithms	was comprised of a total of 247 que
1	algorythms	generation of a 'disposit
1	No mention	No mention
1	No mention	No mention
1	AI algorithms that were	No mention
1	No mention	No mention
1	algorithms	a series of question-
2	artificial intelligence (AI)	it uses a conversational
1	No mention	No mention
are algorithm-based programs that prov	algorythms	No mention
the tools was to be triaging to enhance ·	AI	defined as the theory ar
1	No mention	No mention
1	algorythms	No mention
1	AI	Using machine learning a
3	algorythms	No mention
1	self-diagnosing AI techn	No mention
1	algorythms	apply algorithms to user-answerec
1	algorythms	No mention
	No mention	No mention
1	algorythms	clinical assessment algoı
1	algorythms	Using computerized algc
1	no mention	no mention
1	No mention	no mention
1	No mention	no mention
1	algorythms	no mention
	algorythms	no mention
	algorythms	Static algorithms
1	no mention	no mention
1	no mention	no mention
1	algorythms	recommendation algory
1	no mention	no mention
	no mention	no mention
1	No mention	no mention
1	no mention	no mention
1	algorythm	age and usual health
1	algorythm	no mention
1	algorythms	Using computerized algc
1	algorythm	no mention
1	no mention	no mention

	1	no mention	no mention
	1	no mention	no mention
	1	no mention	no mention
	1	no mention	no mention
	1	algorythm	The algorithm used in th
	1	algorythm	The algorithm used in th
	1	no mention	no mention
	1	no mention	no mention
	2	no mention	no mention
	1	no mention	no mention
	1	algorythm (?)	The NHS symptom checl
	1	algorythms	proprietary diagnostic al
	1	no mention	no mention
	1	no mention	no mention
		no mention	no mention
		no mention	no mention
	1	algorythm	no mention
) apps are health-related applications tha		no mention	no mention
	1	no mention	no mention
	1	algorythms	no mention
algorithms to help patients with self diag		algorythms	Using computerized algo
	2	no mention	no mention
		algorythms	diagnostic algorythms
are algorithm-based programs that prov		algorythms	No mention
	1	No mention	No mention
		algorythms	computerized algorythm
	1	algorythm (?)	The NHS symptom checl
oftware tools that allow users to submit		algorythm (?)	diagnostic algorythms
		use a range of	no mention
octors, CDDS called symptom checkers a		no mention	no mention
		Artificial intelligence pov	no mention
		artificial intelligence algo	The functioning of the medical chatbot is
	1	algorythms	apply algorithms to user-answerec
	1	no mention	no mention
	1	no mention	no mention
	1	no mention	no mention
		algorythm	no mention
website was to provide reliable and unde		no mention	no mention
	1	algorythms	Using computerized algo
	1	algorythm	no mention
	1	algorythm	no mention
		no mention	no mention

	1	No mention	no mention
	1	no mention	no mention
	1	algorythms	Using computerized algo
w or multiple-choice format to ask patie		no mention	no mention
possible implementation of Artificial Int		AI	some level of AI involver
	3	algorythms	No mention
	1	no mention	no mention
		AI powered machine lea	dialogue is enabled by
		Artificial intelligence pov	no mention
		machine learning	labeled automated patie
		algorythms	diagnostic algorythms
	1	algorythm	The algorithm used in th
	1	algorythm	The algorithm used in th
	1	algorythms	proprietary diagnostic al
line screening questionnaire which scree		no mention	no mention
	1	no mention	no mention
d Depression and Anxiety Test (WB-DAT)		algorythms	diagnostic algorythms
		no mention	no mention
	2	no mention	no mention
		no mention	no mention
		Natural Language proces	symptom-diagnosis Kno
gnosis technique that exploits automatic		artificial intelligence algo	a knowledge model
		no mention	no mention
visitor for risk factor information and the		algorythms	no mention
		algorythms	no mention
ator users were based on a set of demog		Monte Carlo model (?)	no mention
curate information about their risk, and,		Monte Carlo model (?)	no mention
sociated with a lesion, the algorithm qua		algorythms	an algorithm for the risk
	1	no mention	no mention
	1	No mention	no mention
risk stratification for lesions analysed		algorythm	hese apps (melanoma ap
	1	no mention	no mention
	1	algorythm (?)	The NHS symptom check
	1	algorythm	age and usual health
	1	algorythms	Using computerized algo
		Bayesian optimization	elicitation is based on
are: by generating data, by looking for pe		no mention	no mention
ker is a smartphone app or web-based fo		AI	Symptom checkers clain
octors, CDDS called symptom checkers a		no mention	no mention
	1	algorythm	no mention
	1	no mention	no mention
	1	algorythm (?)	The NHS symptom check

… are algorithm-based programs that prov		algorythms	No mention
	1	algorythms	No mention
	1	algorythms	no mention
	1	algorythms	apply algorithms to user-answerec
	1	self-diagnosing AI techn	No mention
	3	algorythms	No mention
	1	algorythms	Algorithm-assisted symptom check
	2	no mention	no mention
…) apps are health-related applications tha		no mention	no mention
		AI chatbot	no mention
	1	algorythms	Using computerized algo
		algorythms	triage algorythms
	1	no mention	no mention
		machine learning	As such, many use-
…omponent of mHealth [20, 21]. mHealth		AI	no mention
		Artificial intelligence pov	no mention
	1	AI	Using machine learning a
…'s [5]. Put simply, a patient enters the syr		AI algorythms	These software tools rel
	1	no mention	no mention
	1	No mention	no mention
		algorythms	rule-based algorithms tc
	1	algorythms	recommendation algory
	1	no mention	no mention
…cker apps targeted at specific symptoms		algorythms	diagnostic apps use algo
… conversational agents, interactive agent		AI	no mention
	3	algorythms	No mention
		AI	no mention
		AI	self-learning system (?)
		AI	natural language
	1	algorythms	Using computerized algo
	1	algorythm	no mention
	1	no mention	no mention
…e possible implementation of Artificial Int		AI	some level of AI involver
	1	algorythms	Algorithm-assisted symptom check
…hatbots or virtual assistants, are a type c		AI	no mention
…omponent of mHealth [20, 21]. mHealth		AI	no mention
	1	algorythm (?)	The NHS symptom checl
	1	algorythm	The algorithm used in th
	1	algorythm	The algorithm used in th

	2	no mention	no mention
could improve this situation. SCs are pa		algorythm	background algorithm c
	1	algorithms	a series of question-
	1	algorythms	No mention
ent systems, such as health chatbots, ha		AI	DoctorBot is an AI-drive
	1	algorythm	age and usual health
	1	AI algorithms that were	No mention
	1	No mention	No mention
		no mention	no mention
	1	algorythms	recommendation algory
		no mention	no mention
ool to provide information to adults seek		algorythm	the current COVID-19 al
to self-assess the symptoms of depressi		no mention	no mention
toms reported to GPs by patients befor		algorithms	risk algorithms to calcul
		AI	machine learning
	1	explainable AI	explainable AI, which fo
		no mention	no mention
		AI	information like human
		algorithms and AI (?)	no mention
		no mention	no mention
s [5]. Put simply, a patient enters the sy		AI algorithms	These software tools rel
	1	AI	Using machine learning
		no mention	no mention
		no mention	no mention
	1	No mention	no mention
	1	no mention	no mention
	1	no mention	no mention
		no mention	no mention
	1	algorythms	Using computerized alg
	1	no mention	no mention
	1	algorythm	no mention
	1	algorythms	No mention
		algorythms	diagnostic algorythms
		use a range of	no mention
are algorithm-based programs that pro		algorythms	No mention
	1	No mention	No mention
nptom checkers require the user to resp		AI algorithms and chatb	no mention
	1	Computerized algorithms	was comprised of a total of 247 que
application that patients may utilize to h		AI	MayaMD uses a combin
		algorithms	no mention
		algorithm	The algorithmic questio
	1		
	1	self-diagnosing AI techn	No mention

terms used: AI variable	notes	Amy's "Is i
no mention	/	maybe
no mention	/	maybe
no mention	/	Y
	/	maybe
no mention	/	N
no mention	/	N
no mention	/	maybe
no mention	/	maybe
AI algorithms (?)	/	Y
No mention	/	maybe
No mention	/	maybe
conversational chatbot-style interfac	/	Y
No mention	/	maybe
No mention	/	maybe
No mention	/	Y
No mention	/	maybe
No mention	/	maybe
machine learning natural language pr	/	Y
No mention	/	maybe
No mention	/	Y
No mention	/	Y
No mention	/	maybe
	/	maybe
No mention	/	maybe
no mention	/	maybe
branching logic, bayesian inference, c	/	maybe
no mention	/	maybe
no mention	/	maybe
no mention	/	maybe
no mention	/	maybe
	/	maybe
no mention	/	maybe
no mention	/	N
no mention	/	maybe
no mention	/	maybe
no mention	/	Y
no mention	/	maybe
no mention	/	maybe
no mention	/	maybe
no mention	/	maybe
CDC/AAP clinical algorithm	/	N
no mention	/	maybe
branching logic, bayesian inference, c	/	maybe
	/	maybe
no mention	/	maybe
no mention	/	maybe

no mention	/	maybe
no mention	/	maybe
	/	maybe
no mention	/	maybe
no mention	/	maybe
no mention	/	N
no mention	/	N
no mention	/	maybe
no mention	/	maybe
no mention	/	maybe
	/	maybe
no mention	/	maybe
no mention	/	N
no mention	/	maybe
no mention	/	maybe
no mention	/	maybe
no mention	/	maybe
no mention	/	maybe
	/	maybe
no mention	/	maybe
no mention	/	maybe
no mention	/	maybe
no mention	/	maybe
	/	maybe
The algorithms vary and may use bra	/	maybe
no mention	/	maybe
no mention	/	maybe
No mention	/	maybe
	/	maybe
no mention	/	maybe
Through artificial intelligence algorithms that guide the patient’s	/	Y
no mention	/	N
no mention	/	maybe
no mention	/	maybe
no mention	/	maybe
	/	maybe
no mention	/	Y
natural language processing, machine	/	Y
No mention	/	Y
no mention	/	maybe
no mention	/	maybe
no mention		maybe
no mention		maybe
no mention		maybe
branching logic, bayesian inference, c	/	maybe
no mention	/	maybe
no mention	/	maybe
no mention		maybe

no mention	/	maybe
no mention	/	maybe
		maybe
branching logic, bayesian inference, c	/	maybe
these software tools use a range of c	1st author's affiliation with ADA	maybe
no mention		Y
No mention	/	maybe
no mention	/	maybe
employs a finite state model for	1st author's affiliation with Medius Health	Y
no mention	/	Y
a naïve Bayes triage model was creat	/	Y
no mention	/	maybe
no mention	/	N
no mention	/	N
no mention	/	maybe
no mention		maybe
no mention	/	maybe
no mention		maybe
no mention		maybe
no mention	/	maybe
no mention		N
no mention	1st author's affiliation with Medius Health	Y
ranking methods	/	Y
no mention	/	maybe
no mention	/	maybe
no mention	/	maybe
no mention	/	N
no mention	/	N
fractal analysis	/	Y
no mention	/	maybe
no mention	/	maybe
no mention	/	Y
no mention	/	maybe
no mention	/	N
CDC/AAP clinical algorithm	/	N
branching logic, bayesian inference, c	/	maybe
learning model (?)	/	Y
	/	maybe
	/	maybe
no mention	/	maybe
no mention	/	maybe
no mention	/	maybe
no mention	/	maybe
no mention	/	maybe
	/	maybe
	/	maybe
no mention	/	N

	/	maybe
	/	maybe
No mention	/	maybe
No mention	/	maybe
no mention	/	maybe
No mention	/	Y
No mention	/	Y
No mention	/	maybe
no mention	/	maybe
no mention	/	maybe
no mention	/	maybe
	/	maybe
machine learning (?)	/	Y
branching logic, bayesian inference, c	/	maybe
no mention	/	maybe
no mention	/	maybe
machine learning/ control algorithm	/	Y
machine learning	/	Y
no mention	/	Y
machine learning natural language pr	/	Y
various	1st author's affiliation with ADA	Y
		maybe
no mention	/	maybe
no mention	/	maybe
	/	maybe
derived from a diagnostic and		maybe
no mention	/	Y
no mention	/	maybe
		maybe
no mention		Y
no mention		maybe
No mention	/	maybe
machine learning		Y
The knowledge base was created		Y
natural language processing, machine learning		Y
branching logic, bayesian inference, c	/	maybe
no mention	/	maybe
no mention	/	maybe
no mention		Y
no mention	/	maybe
machine learning		Y
machine learning	/	Y
	/	maybe
		maybe
	/	maybe
no mention	/	N
no mention	/	N
no mention	/	N

no mention	/	maybe
no mention		Y
No mention	/	maybe
No mention	/	maybe
deep learning and knowledge graphs	/	Y
CDC/AAP clinical algorithm	/	N
AI algorithms (?)	/	Y
No mention	/	maybe
no mention		maybe
no mention	/	maybe
no mention		maybe
self-triage algorithm		N
no mention		maybe
no mention		N
on fixed rules, non-neural		Y
no mention	/	Y
no mention		N
no mention		Y
explainable artificial intelligence ?		Y
no mention		Y
various	1st author's affiliation with ADA	Y
machine learning natural language pr	/	Y
no mention	1st author's affiliation with tool company	maybe
no mention		maybe
no mention	/	maybe
no mention	/	maybe
no mention	/	maybe
no mention		maybe
branching logic, bayesian inference, c	/	maybe
no mention	/	maybe
no mention	/	maybe
No mention	/	maybe
no mention	/	maybe
no mention	/	maybe
No mention	/	maybe
no mention	/	maybe
no mention		Y
no mention	/	N
bayesian statistics and machine learn	/	Y
no mention	1st author's affiliation with ADA	Y
no mention		maybe
	/	maybe
No mention	/	Y

t actually talking about AI?" question Y/N/maybe
